# Synthesis of 4-(Phenylchalcogenyl)tetrazolo[1,5-*a*]quinolines by Bicyclization of 2-Azidobenzaldehydes with Phenylchalcogenylacetonitrile

**DOI:** 10.3390/molecules28135036

**Published:** 2023-06-27

**Authors:** Loana I. Monzon, Nicole C. M. Rocha, Gabriela T. Quadros, Pâmela P. P. Nunes, Roberta Cargnelutti, Raquel G. Jacob, Eder J. Lenardão, Gelson Perin, Daniela Hartwig

**Affiliations:** 1 Laboratório de Síntese Orgânica Limpa-LaSOL-CCQFA, Universidade Federal de Pelotas—UFPel, P.O. Box 354, Pelotas 96010-900, RS, Brazil; loanamonzon97@gmail.com (L.I.M.); nicolecmrocha@outlook.com (N.C.M.R.); gabrielatrischdequadros@gmail.com (G.T.Q.); pamelapotenza@hotmail.com (P.P.P.N.); lenardao@ufpel.edu.br (E.J.L.); 2 Departamento de Química, Universidade Federal de Santa Maria—UFSM, Av. Roraima, Building 18, Santa Maria 97105-900, RS, Brazil; roberta.cargnelutti@ufsm.br

**Keywords:** organochalcogen, tetrazolequinolines, cyclization, heterocycles

## Abstract

A general methodology to access valuable 4-(phenylchalcogenyl)tetrazolo[1,5-*a*]quinolines was developed by the reaction of 2-azidobenzaldehyde with phenylchalcogenylacetonitriles (sulfur and selenium) in the presence of potassium carbonate (20 mol%) as a catalyst. The reactions were conducted using a mixture of dimethylsulfoxide and water (7:3) as solvent at 80 °C for 4 h. This new methodology presents a good functional group tolerance to electron-deficient and electron-rich substituents, affording a total of twelve different 4-(phenylchalcogenyl)tetrazolo[1,5-*a*]quinolines selectively in moderate to excellent yields. The structure of the synthesized 4-(phenylselanyl)tetrazolo[1,5-*a*]quinoline was confirmed by X-ray analysis.

## 1. Introduction

**Figure 1:**
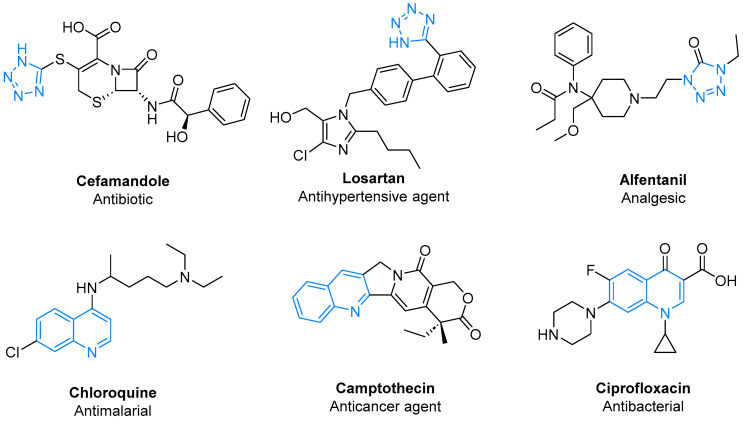
Commercially available drugs containing the tetrazole or quinoline moiety.

Tetrazoles and quinolines are members of the important class of the azaheterocycles, which have a wide variety of applications. The first ones consist in a five-membered ring with four nitrogen and one carbon atom. Even though they cannot be found in nature, tetrazoles are very stable considering the number of nitrogen atoms. Tetrazole derivatives are used as explosives and fuels in the arms industry and as agrochemicals, and they can be found in over 40 commercial drugs as antibiotic, antiallergic, analgesic, and antihypertensive agents (Figure 1) [1,2,3,4].

On the other hand, quinoline scaffold is a benzene ring-fused pyridine mostly found in various natural products, particularly in alkaloids. Quinolines have multipurpose applications in medicinal chemistry, such as anticancer, antibacterial, antifungal, anthelmintic, antiprotozoal, anticonvulsant, antitubercular, anti-inflammatory, analgesic, antimalarials, and antiviral agents (Figure 1) [5,6,7].

Tetrazoloquinolines are molecular hybrids that contain both quinoline and tetrazole systems in the same molecule, and they have received much attention due to their biological activities and synthetic usefulness [8,9,10,11]. Despite the significant advances toward the synthesis of new azaheterocycles, the need for a new study on the combinations of substrates for the synthesis of new tetrazoloquinolines and more complex structures is still an open issue. 

In this context, chalcogen–tetrazolo[1,5-*a*]quinolines constitute an interesting class of molecules, which combine the importance of a tetrazoloquinoline nucleus [8,9,10,11] with an organochalcogen moiety [12,13,14]. Selenium and sulfur are important essential elements, playing important roles in metabolic pathways [15,16,17,18,19,20], and the interest in chalcogen chemistry [21,22,23] and pharmacology [24,25,26,27] has increased in this century.

**Scheme 1:**
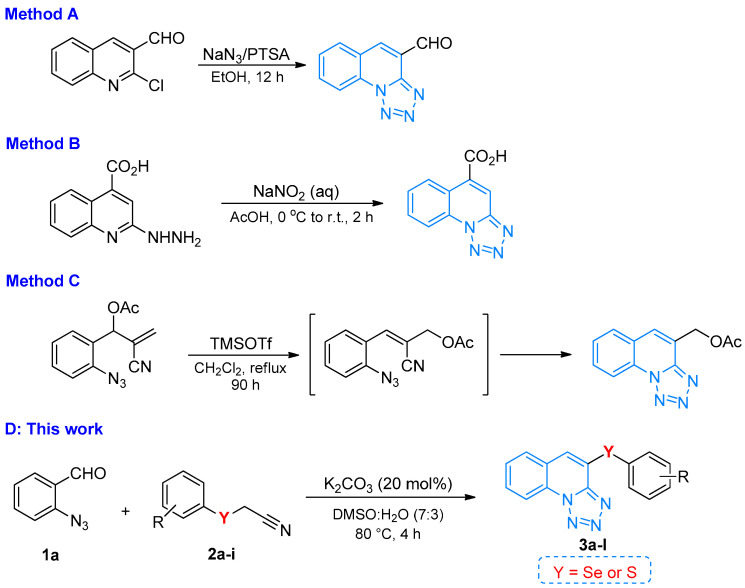
Previous methods to prepare tetrazolo[1,5-*a*]quinolines. Method A: from 2-chloroquinoline and NaN_3_ [32]; Method B: from 2-hydrazinylquinoline [29]; Method C: from 2-azidoarylidenes [31,34]; Method D: this work.

Several methodologies [28,29,30,31,32,33,34] have been reported for the synthesis of a range of tetrazolo[1,5-*a*]quinolines such as (i) from the reaction of 2-chloroquinoline with sodium azide (Scheme 1, Method A) [32]; (ii) from the diazotization of 2-hydrazinylquinoline derivatives (Scheme 1, Method B) [29]; and (iii) from the intramolecular cyclocondensation of 2-azidoarylidenes (Scheme 1, Method C) [31,34]. However, to the best of our knowledge, there are no procedures to directly prepare 4-(phenylchalcogenyl)tetrazol[1,5-*a*]quinolines. Additionally, most of the described synthetic routes to obtain tetrazolo[1,5-*a*]quinolines involve high temperatures, long reaction times, harsh catalysis, and toxic solvents [28]. 

Tetrazole or triazole nucleus can be formed in very good yields through the fast, concerted and regioselective [3 + 2] cycloaddition between arylazides and acetonitrile-bearing active CH groups [33,34,35,36]. Heating is necessary for poorly reactive substrates, but if strong electron-withdrawing groups are present in the acetonitrile counterpart, the reaction takes place rapidly. In the same way, the intramolecular cycloaddition can occur quickly in molecules that have these two groups (nitrile and azide) in the same chain [33,34,35]. 

**Scheme 2:**
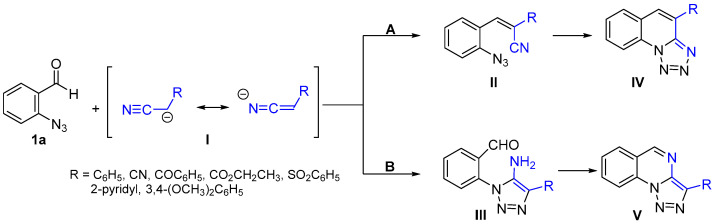
Reaction pathways of the cyclization starting from 2-azidobenzaldehyde **1a**. Path A: the first step is a Knoevenagel condensation to form **II**, followed by the cyclization to tetrazole **IV**. Path B: the first step is the formation of the triazole **III**, followed by the cyclization to quinazoline **V**.

Polycyclic tetrazoles can be obtained when various arylazides containing an electrophilic group at the *ortho* position are used. Thus, cyclocondensation of 2-azidobenzaldehydes with acetonitrile derivatives under base catalysis have been used to produce tetrazolo[1,5-*a*]quinoline derivatives [28]. However, with 2-azidobenzaldehyde **1a**, the active methylene anion of acetonitrile **I** reacts with both the aldehyde and azide sites, resulting in two reaction pathways (Scheme 2) [34]. Subsequent cyclization of the respective intermediates **II** and **III** formed in situ leads to two different heterocyclic compounds. The pathway cyclization depends on the reaction conditions and are subject to solvent effects [34]. Usually, in the presence of protic solvents and heating, tetrazolo[1,5-*a*]quinolines **IV** were synthesized, whereas in aprotic solvent and at low temperature, 1,2,3-triazolo[1,5-*a*]quinazolines **V** were obtained [34,35,36]. 

In path **A**, the first step of the reaction involves the Knoevenagel condensation of aldehyde with the methylene anion of acetonitrile, formed in the presence of a base. In the second step, the intramolecular [3 + 2] cycloaddition of **II** occurs, affording the tetrazolo[1,5-*a*]quinoline **IV**. Pathway **B**, instead, starts with the attack of acetonitrile carbanion on the azide function followed by the intramolecular cyclocondensation between the amino and carbonyl groups of the aminotriazole intermediate **III**, affording the 1,2,3-triazolo[1,5-*a*]quinazoline **V** (Scheme 2).

Based on what was described so far, and in our interest in developing new strategies to prepare potentially bioactive organochalcogen compounds, we decided to explore the reaction of Scheme 2 to create so far unprecedented hybrid molecules containing both the tetrazolo[1,5-*a*]quinoline and organochalcogen moieties under green conditions. Therefore, herein we describe the synthesis of 4-(arylchalcogenyl)tetrazolo[1,5-*a*]quinoline **3a–l** through the intramolecular cyclocondensation between 2-azidobenzaldehydes **1** and appropriate 2-(arylchalcogenyl)acetonitriles **2**, using K_2_CO_3_ as base in a mixture of DMSO:H_2_O as solvent (Scheme 1, Method D). 

## 2. Results and Discussion

Initially, we chose 2-azidobenzaldehyde **1a** and 2-(arylselanyl)acetonitrile **2a** as model substrates to establish the best conditions for this reaction, and some experiments were performed to synthesize the corresponding tetrazolo[1,5-*a*]quinoline **3a** (Table 1). We started our studies by reacting **1a** (0.25 mmol) with **2a** (0.25 mmol) at 80 °C for 4 h, using K_2_CO_3_ (20 mol%) as a catalyst in 1.0 mL of DMSO as solvent. Under these conditions, the desired product **3a** was obtained in 21% yield (Table 1, entry 1). When water was used as the solvent, only traces of **3a** were detected by GC/MS (Table 1, entry 2). An excellent result was obtained when a mixture of DMSO:H_2_O (7:3) was used as solvent, leading to the expected product **3a** in 98% yield (Table 1, entry 3), showing that a small amount of water is needed to dissolve the potassium carbonate. 

Next, we investigated the use of different solvents; water was mixed with glycerol, DMF, toluene and acetonitrile. Unfortunately, a decrease in the yield of **3a** was observed in all cases, with glycerol being the best in class (Table 1, entries 4–7). When the reaction was performed without solvent, the expected product **3a** was obtained in only 10% yield (Table 1, entry 8). Hence, a 7:3 mixture of DMSO and water was chosen as the best solvent for this reaction.

As a way to verify how different bases can affect the reaction, cesium carbonate (Cs_2_CO_3_), potassium hydroxide (KOH), triethylamine (Et_3_N), and 1,8-diazobicyclo[5.4.0]undec-7-ene (DBU) were tested in the reaction of **1a** with **2a** (Table 1, entries 9–12). However, except for Cs_2_CO_3_ (92% yield), the observed results were not satisfactory, and lower yields of **3a** were obtained in all cases. When the amount of K_2_CO_3_ was reduced to 10 mol%, the desired product **3a** was obtained in only 10% yield, while no product was observed in the absence of base, demonstrating the crucial role of the base (Table 1, entries 13 and 14). Thus, K_2_CO_3_ that is considered a green catalyst, [37] was set as the best base for this reaction. When the reactions were performed at 60 °C and 100 °C, the desired product **3a** was obtained in 30% and 62% yield, respectively (Table 1, entries 15 and 16). The tetrazolo[1,5-*a*]quinoline **3a** was respectively obtained in 68% and 22% yield after 3 and 2 h of reaction (Table 1, entries 17 and 18). Finally, a reaction was performed at room temperature, to verify the possibility of preparing the respective 1,2,3-triazolo[1,5-*a*]quinazoline **V** (see Scheme 2, Pathway **B**). After 4 h of reaction, only 50% of the starting aldehyde was consumed, and a mixture of triazole **8a** and tetrazole derivative **3a** was obtained in a 2:8 ratio (Table 1, entry 19).

Based on the results shown in Table 1, the best reaction conditions involve stirring a mixture of 2-azidobenzaldehyde (**1a**, 0.25 mmol) and 2-(arylselanyl)acetonitrile (**2a**, 0.25 mmol) in the presence of K_2_CO_3_ (20 mol%) as base in a 7:3 mixture of DMSO:H_2_O (1.0 mL) as solvent for 4 h at 80 °C.

**Figure 2:**
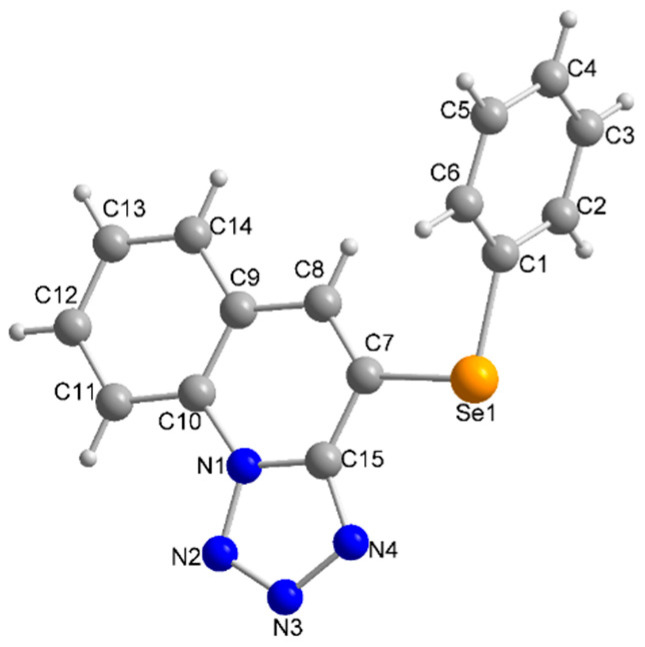
Projection of the molecular structure of 4-(phenylselanyl)tetrazolo[1,5-*a*]quinoline (**3a**) obtained by X-ray crystallography.

The identity of the new 4-(arylchalcogenyl)tetrazolo[1,5-*a*]quinolines was confirmed by HRMS, and ^1^H, ^13^C and ^77^Se-NMR analysis. In the ^1^H-NMR spectrum of all the prepared compounds, a characteristic signal is a doublet (*J* ≅ 8.0 Hz) around 8.5 ppm, referring to H-6, while in the ^13^C-NMR the characteristic tetrazole ring C_sp2_ appears at 146.5–147.0 ppm. In addition, the chemical structure of 4-(phenylselanyl)tetrazolo[1,5-*a*]quinoline **3a** was confirmed by a single crystal X-ray diffraction analysis (Figure 2) (for details, see the Supplementary Materials). Colorless crystals of compound **3a** were obtained by the slow evaporation of the dichloromethane/ethyl acetate (1:1) mixture, in the refrigerator. The obtained crystals were analyzed by single crystal X-ray diffraction analysis (Figure 2). The distance between Se(1)–C(1) and Se(1)–C(7) is 1.916(2) and 1.899(2) Å, respectively. The N–N and N–C distance in the tetrazol portion of the structure is N(1)–N(2) 1.364(3); N(3)–N(2) 1.304(4), N(3)–N(4) 1.358(4); N(1)–C(15) 1.349(3) and N(4)–C(15) 1.327(3) Å. The angle between the C(7)–Se(1)–C(1) is 100.23(9)°.

**Scheme 3:**
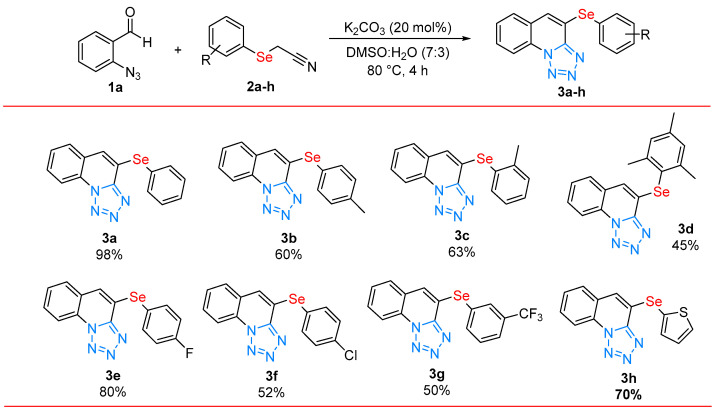
4-(Arylselanyl)tetrazolo[1,5-*a*]quinolines **3a–h**: scope of 2-(arylselanyl)acetonitriles **2**.

The scope of the proposed methodology was then extended to differently substituted arylselanyl-acetonitrile **2a–h**, in the reaction with 2-azidobenzaldehyde **1a**, aiming to investigate the generality and limitations of the method (Scheme 3). Interestingly, the presence of substituents in the benzene ring of the arylselanyl-acetonitrile counterpart decreases the reactivity compared to the unsubstituted **2a**, but a clear electronic effect could not be observed. For instance, electron-rich 2-(*p*-tolylselanyl)acetonitrile **2b**, 2-(*o*-tolylselanyl)acetonitrile **2c**, and 2-(mesitylselanyl)acetonitrile **2d** reacted smoothly with **1a** to afford the respective tetrazolo[1,5-*a*]quinolines **3b**, **3c**, and **3d** in 60%, 63%, and 45% yield. Similarly, the electron-poor 2-((4-chlorophenyl)selanyl)acetonitrile **2f** and 2-((3-(trifluoromethyl)phenyl)selanyl)acetonitrile **2g** produced the expected products **3f** and **3g** in 52% and 50% yield, respectively. A remarkable result was obtained in the reaction of 2-((4-fluorophenyl)selanyl)acetonitrile **2e**, which afforded 4-((4-fluorophenyl)selanyl)tetrazolo[1,5-*a*]quinoline **3e** in 80% yield under optimal conditions. Satisfactorily, the protocol was suitable for the heteroaromatic derivative **2h**, and 4-(thiophen-2-ylselanyl)tetrazolo[1,5-*a*]quinoline **3h** was synthesized in 70% yield.

**Scheme 4:**
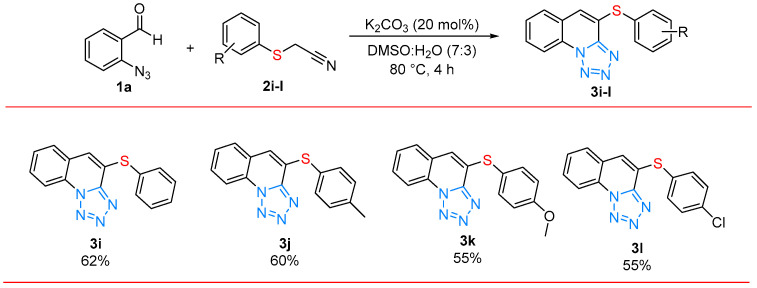
4-(Arylthio)tetrazolo[1,5-*a*]quinolines **3i–l**: scope of 2-(arylthio)acetonitriles **2**.

Subsequently, we investigated the reactivity of a variety of arylthioacetonitriles **2i–l** with 2-azidobenzaldehyde **1a** under the optimized conditions (Scheme 4). In contrast to the observed for the selenium analogs, the presence of substituents in *para*-position of the benzene ring of the thioacetonitrile did not strongly influence the reactivity. Thus, unsubstituted 2-(phenylthio)acetonitrile **2i** afforded **3i** in 62% yield, while the substituted derivatives **3j**, **3k**, and **3l** were obtained in 60%, 55%, and 55% yield, respectively.

**Scheme 5:**
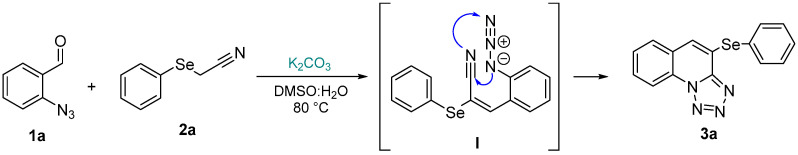
Plausible mechanism.

Based on the literature [33,34,35,36] and our own results, we believe that the reaction probably proceeds via path A (Scheme 2), suggesting that the plausive mechanism for the formation of 4-(arylchalcogenyl)tetrazolo[1,5-*a*]quinoline **3** is that of Scheme 5. Initially, in the presence of K_2_CO_3_, the Knoevenagel condensation between 2-azidobenzaldehyde **1a** and 2-(phenylselanyl)-acetonirile **2a** occurs, leading to the intermediate **I**. Then, an intramolecular [3 + 2] cycloaddition of the azido group to the nitrile group in intermediate **I** furnishes the 4-(phenylchalcogenyl)tetrazolo[1,5-*a*]quinoline **3a**. 

## 3. Materials and Methods

### 3.1. General Information

Reactions were carried out in a two-necked round-bottomed flask with a Teflon-coated magnetic stirring bar. Solvents and reagents were used as received unless otherwise noted. The reactions were monitored by thin-layer chromatography (TLC), which was performed using Merck (Merck, Darmstadt, Germany) silica gel (60 F_254_), with a 0.25 mm thickness. For visualization, TLC plates were either exposed to UV light, or stained with iodine vapor or in a 5% vanillin solution in 10% aqueous H_2_SO_4_ and heat. Hydrogen nuclear magnetic resonance spectra (^1^H NMR) were obtained on a Bruker Avance III HD 400 MHz employing a direct broad-band probe at 400 MHz. The spectra were recorded in CDCl_3_ or DMSO-*d*_6_ solutions. The chemical shifts (δ) are reported in ppm, referenced to tetramethylsilane (TMS) as the internal reference. Coupling constants (*J*) are reported in Hertz. Carbon-13 nuclear magnetic resonance spectra (^13^C NMR) were obtained on Bruker Avance III HD 400 MHz employing a direct broad-band probe at 100 MHz. The chemical shifts (δ) are reported in ppm, referenced to the solvent peak of CDCl_3_ (δ 77.0 ppm) or DMSO-*d*_6_ (39.7 ppm). Selenium-77 nuclear magnetic resonance spectra (^77^Se NMR) were obtained on a Bruker Avance III HD 400 MHz employing direct broad-band probe at 76 MHz. The chemical shifts (δ) are reported in ppm, using as solvent CDCl_3_ and diphenyl diselenide as an internal standard (δ 463 ppm). Figures of the NMR spectra are presented in the Supplementary Materials. High-resolution mass spectra (HRMS) were recorded in positive ion mode (APCI) using a Q-TOF or a Quadrupole-Orbitrap spectrometer. Melting point (mp) values were measured in a Marte PFD III instrument with a 0.1 °C precision. The starting 2-azidobenzaldehydes **1** [38] and 2-(arylchalcogenyl)acetonitriles **2a–l** [39] were synthesized according to the literature.

### 3.2. Synthesis of 2-Azidobenzaldehyde ***1a***

2-Azidobenzaldehyde (**1a**) was synthesized according to the methodology proposed by Qiu et al., with some modifications [38]. In a 100 mL two-necked round-bottomed flask equipped with magnetic stirring and at 60 °C (oil bath), 15 mmol (2.26 g) of 2-nitrobenzaldehyde and 25 mL of dimethylformamide (DMF) were added. The solution was stirred for 5 min and after that, 30 mmol (1.95 g) of sodium azide (NaN_3_) was added under air atmosphere. The resulting mixture was stirred at 60 °C for 48 h. After this time, the resulting solution was received into water (150 mL) and the product was extracted with ethyl acetate (3 × 50 mL). The organic layer was separated, dried over anhydrous magnesium sulfate, filtered, and concentrated under reduced pressure (rotary evaporator). The crude oil was purified by column chromatography using silica gel and hexane–ethyl acetate (98:02) as the eluent. The respective product was obtained in 54% of yield (1.19 g).

### 3.3. General Procedure for the Synthesis of (Arylchalcogenyl)acetonitriles ***2a–l***

The (arylchalcogenyl)acetonitriles **2a–l** were synthesized according to the methodology proposed by Alves et al., with some modifications [39]. In a 25 mL round-bottomed reaction flask equipped with magnetic stirring, 1.2 mmol of diaryl chalcogenide (RSeSeR or RSSR), 5 mL of tetrahydrofuran (THF) and 2 mL of ethanol were added. The resulting mixture was stirred for a few minutes at 0 °C under N_2_ atmosphere. Then, 3 mmol (0.11 g) of sodium borohydride was slowly added. After that, the reaction color changed from yellow to white. In the sequence, 2.5 mmol of the respective chloronitrile was added and stirred at room temperature under N_2_ atmosphere for 8 h. After this time, the reaction mixture was received into water (150 mL) and the product was extracted with ethyl acetate (3 × 25 mL). The organic layer was separated, washed with brine (50 mL), dried over anhydrous MgSO_4_, filtered, and concentrated under vacuum (rotary evaporator). The residue was purified by column chromatography using silica gel and hexane–ethyl acetate as the eluent. The products **2a–l** were obtained in good to excellent yields.

### 3.4. General Reaction Procedure for the Synthesis of 4-(Arylchalcogenyl)tetrazolo[1,5-a]quinolines ***3a–l***

2-Azidobenzaldehyde **1a** (0.037 g, 0.25 mmol), 2-(arylchalcogenyl)acetonitrile **2** (0.25 mmol), K_2_CO_3_ (0.007 g, 20 mol%) and DMSO:H_2_O (7:3, 1 mL) were added to a 25 mL two-necked round-bottomed flask. The system was then immersed in a preheated oil bath at 80 °C and stirred at this temperature until total disappearance of the starting materials. Reactions were monitored by thin-layer chromatography (TLC). After the completion of the reaction, the reaction mixture was cooled to room temperature and extracted with EtOAc (3 × 10 mL), dried over MgSO_4_, and the solvent was evaporated under reduced pressure. The residue was purified by preparative chromatographic plate using ethyl acetate/hexane as the eluent. Spectral data for the prepared products are listed below.

4-(Phenylselanyl)tetrazolo[1,5-*a*]quinoline (**3a**): Brown solid, mp: 171–174 °C. Yield: 98%. ^1^H NMR (400 MHz, CDCl_3_) δ: 8.57 (d, *J* = 8.3 Hz, 1H), 7.77 (dd, *J* = 14.9 and 7.5 Hz, 3H), 7.67 (d, *J* = 7.8 Hz, 1H), 7.59 (t, *J* = 7.5 Hz, 1H), 7.49 (dt, *J* = 14.1 and 7.0 Hz, 3H), 7.37 (s, 1H). ^13^C NMR (100 MHz, CDCl_3_) δ: 147.1, 136.5 (2C), 130.9, 130.1 (2C), 130.0, 129.8, 129.2, 127.9, 127.7, 125.2, 124.6, 119.7, 116.6. ^77^Se NMR (76 MHz, CDCl_3_) δ: 397.49. HRMS *m*/*z*: [M + H]^+^ Calcd. for C_15_H_10_N_4_Se: 327.0143. Found: 327.0134.

4-(*p*-Tolylselanyl)tetrazolo[1,5-*a*]quinoline (**3b**): Brown solid, mp: 123–125 °C. Yield: 60%. 

^1^H NMR (400 MHz, CDCl_3_) δ: 8.56 (d, *J* = 8.3 Hz, 1H), 7.74 (t, *J* = 8.3 Hz, 1H), 7.66 (d, *J* = 8.0 Hz, 3H), 7.58 (t, *J* = 7.6 Hz, 1H), 7.31 (s, 1H), 7.27 (d, *J* = 7.9 Hz, 2H), 2.24 (s, 3H). ^13^C NMR (100 MHz, CDCl_3_) δ: 147.0, 140.2, 136.7 (2C), 131.0 (2C), 130.2, 129.8, 129.1, 127.9, 127.6, 124.6, 121.3, 120.4, 116.6, 21.3. ^77^Se NMR (76 MHz, CDCl_3_) δ: 353.31. HRMS *m*/*z*: [M + H]^+^ Calcd. for C_16_H_12_N_4_Se: 341.0300. Found: 341.0290.

4-(*o*-Tolylselanyl)tetrazolo[1,5-*a*]quinoline (**3c**): White solid, mp: 167–169 °C. Yield: 63%. ^1^H NMR (400 MHz, CDCl_3_) δ: 8.56 (d, *J* = 8.3 Hz, 1H), 7.77–7.72 (m, 2H), 7.65 (d, *J* = 7.4 Hz, 1H), 7.58 (t, *J* = 7.5 Hz, 1H), 7.43 (d, *J* = 4.2 Hz, 2H), 7.28–7.24 (m, 1H), 7.14 (s, 1H), 2.51 (s, 3H). ^13^C NMR (100 MHz, CDCl_3_) δ: 147.0, 142.9, 137.9, 130.9, 130.5, 129.8, 129.1, 129.0, 127.9, 127.6, 127.5, 125.7, 124.6, 119.3, 116.5, 22.7. ^77^Se NMR (76 MHz, CDCl_3_) δ: 354.05. HRMS *m*/*z*: [M + H]^+^ Calcd. for C_16_H_12_N_4_Se: 341.0300. Found: 341.0287.

4-(Mesitylselanyl)tetrazolo[1,5-*a*]quinoline (**3d**): White solid, mp: 184–186 °C. Yield: 45%. ^1^H NMR (400 MHz, CDCl_3_) δ: 8.49 (d, *J* = 8.1 Hz, 1H), 7.64 (t, *J* = 7.3 Hz, 1H), 7.55 (d, *J* = 7.5 Hz, 1H), 7.48 (t, *J* = 7.2 Hz, 1H), 7.03 (s, 2H), 6.83 (s, 1H), 2.41 (s, 6H), 2.31 (s, 3H). ^13^C NMR (100 MHz, CDCl_3_) δ: 147.09, 144.1 (2C), 140.61, 129.4 (2C), 129.38, 128.89, 127.80, 127.44, 127.08, 124.89, 122.94, 119.82, 116.61, 23.9 (2C), 21.15. ^77^Se NMR (76 MHz, CDCl_3_) δ: 280.22. HRMS *m*/*z*: [M + H]^+^ Calcd. for C_18_H_16_N_4_Se: 369.0613. Found: 369.0608.

4-((4-Fluorophenyl)selanyl)tetrazolo[1,5-*a*]quinoline (**3e**): Yellow solid, mp: 163–167 °C. Yield: 80%. ^1^H NMR (400 MHz, CDCl_3_) δ: 8.55 (d, *J* = 8.3 Hz, 1H), 7.79–7.72 (m, 3H), 7.66 (d, *J* = 7.9 Hz, 1H), 7.58 (t, *J* = 8.1 Hz, 1H), 7.52–7.44 (m, 2H), 7.36 (s, 1H). ^13^C NMR (100 MHz, CDCl_3_) δ: 163.8 (d, *J* = 251.8 Hz), 146.3, 137.4 (d, *J* = 8.6 Hz, 2C), 130.1, 129.0, 128.1, 127.8, 127.8, 125.4, 124.3, 124.0 (d, *J* = 3.4 Hz), 117.4 (d, *J* = 22.1 Hz, 2C), 116.7. ^77^Se NMR (76 MHz, CDCl_3_) δ: 388.82. HRMS *m*/*z*: [M + H]^+^ Calcd. for C_15_H_9_FN_4_Se: 345.0049. Found: 345.0052.

4-((4-Chlorophenyl)selanyl)tetrazolo[1,5-*a*]quinoline (**3f**): White solid, mp: 146–149 °C. Yield: 52%. ^1^H NMR (400 MHz, CDCl_3_) δ: 8.58 (d, *J* = 8.3 Hz, 1H), 7.79 (t, *J* = 8.3 Hz, 1H), 7.71 (d, *J* = 8.5 Hz, 3H), 7.63 (t, *J* = 7.6 Hz, 1H), 7.45 (s, 1H), 7.41 (d, *J* = 8.4 Hz, 2H). ^13^C NMR (100 MHz, CDCl_3_) δ: 147.1, 137.6 (2C), 136.2, 131.6, 130.4, 130.3 (2C), 129.4, 128.1, 127.9, 124.4, 123.6, 118.8, 116.7. ^77^Se NMR (76 MHz, CDCl_3_) δ: 391.28. HRMS *m*/*z*: [M + H]^+^ Calcd. for C_15_H_9_ClN_4_Se: 360.9754. Found: 360.9735.

4-((3-(Trifluoromethyl)phenyl)selanyl)tetrazolo[1,5-*a*]quinoline (**3g**): Yellow solid, mp: 145–149 °C. Yield: 50%. ^1^H NMR (400 MHz, CDCl_3_) δ: 8.62 (d, *J* = 8.3 Hz, 1H), 8.03 (s, 1H), 7.97 (dd, *J* = 7.8 Hz, 1H), 7.84 (td, *J* = 7.3 Hz, 1H), 7.77 (d, *J* = 8.0 Hz, 1H), 7.73–7.71 (m, 1H), 7.66 (td, *J* = 8.6 Hz, 1H), 7.58 (s, 1H), 7.56 (d, *J* = 8.1 Hz, 1H). ^13^C NMR (100 MHz, CDCl_3_) δ: 147.3, 139.2, 133.1, 132.3 (q, *J* = 3.8 Hz), 132.2 (q, *J* = 32.8 Hz), 130.8, 130.4, 128.7, 128.4 (q, *J* = 266.0 Hz), 128.2, 128.1, 126.3 (q, *J* = 3.8 Hz), 124.7, 122.0, 117.6, 116.8. ^77^Se NMR (76 MHz, CDCl_3_) δ: 400.57. HRMS *m*/*z*: [M + H]^+^ Calcd. for C_16_H_9_F_3_N_4_Se: 395.0017. Found: 395.0017.

4-(Thiophen-2-ylselanyl)tetrazolo[1,5-*a*]quinoline (**3h**): White solid, mp: 160–163 °C. Yield: 70%. ^1^H NMR (400 MHz, CDCl_3_) δ: 8.54 (d, *J* = 8.3 Hz, 1H), 7.75 (td, *J* = 8.4 and 1.3 Hz, 1H), 7.70–7.66 (m, 2H), 7.59 (td, *J* = 8.2 and 1.2 Hz, 1H), 7.55 (dd, *J* = 3.5 and 1.2 Hz, 1H), 7.31 (s, 1H), 7.23–7.21 (m, 1H). ^13^C NMR (100 MHz, CDCl_3_) δ: 146.5, 139.4, 134.1, 130.10, 130.09, 129.1, 129.0, 127.9, 127.8, 124,5, 120.4, 117.9, 116.5. HRMS *m/z*: [M + H]^+^ Calcd. for C_13_H_8_N_4_SSe: 332.9708. Found: 332.9701.

4-(Phenylthio)tetrazolo[1,5-*a*]quinoline (**3i**): Brown solid, mp: 175–178 °C. Yield: 62%. ^1^H NMR (400 MHz, CDCl_3_) δ: 8.57 (d, *J* = 8.3 Hz, 1H), 7.75 (t, *J* = 8.3 Hz, 1H), 7.70–7.66 (m, 3H), 7.60 (t, *J* = 7.6 Hz, 1H), 7.51–7.49 (m, 3H), 7.24 (s, 1H). ^13^C NMR (100 MHz, CDCl_3_) δ: 146.4, 134.9 (2C), 130.1 (2C), 129.9, 129.9, 128.9, 128.8, 128.0, 127.8 (2C), 125.4, 124.3, 116.6. HRMS *m/z*: [M + H]^+^ Calcd. for C_15_H_10_N_4_S: 279.0699. Found: 279.0692.

4-(*p*-Tolylthio)tetrazolo[1,5-*a*]quinoline (**3j**): Brown solid, mp: 122–126 °C. Yield: 60%. ^1^H NMR (400 MHz, DMSO-*d*_6_) δ: 8.67 (d, *J* = 8.3 Hz, 1H), 8.19 (d, *J* = 7.9 Hz, 1H), 8.02 (t, *J* = 7.6 Hz, 1H), 7.87–7.83 (m, 2H), 7.66 (d, *J* = 8.0 Hz, 2H), 7.46 (d, *J* = 7.9 Hz, 2H), 3.47 (s, 3H). ^13^C NMR (100 MHz, DMSO) δ: 146.3, 139.2, 133.4 (2C), 130.8 (2C), 130.7, 130.1, 128.8, 128.6, 128.3, 125.8, 124.1, 122.8, 116.1, 20.8. HRMS *m*/*z*: [M + H]^+^ Calcd. for C_16_H_12_N_4_S: 293.0855. Found: 293.0851.

4-((4-Methoxyphenyl)thio)tetrazolo[1,5-*a*]quinoline (**3k**): White solid, mp: 157–159 °C. Yield: 55%. ^1^H NMR (400 MHz, CDCl_3_) δ: 8.55 (d, *J* = 8.3 Hz, 1H), 7.71 (t, *J* = 7.7 Hz, 1H), 7.67–7.56 (m, 4H), 7.10–7.01 (m, 3H), 3.90 (s, 3H). ^13^C NMR (100 MHz, CDCl_3_) δ: 161.3, 146.1, 137.4 (2C), 129.5, 128.6, 127.9, 127.6, 127.1, 125.9, 124.4, 118.3, 116.5, 115.7 (2C), 55.5. HRMS *m*/*z*: [M + H]^+^ Calcd. for C_16_H_12_N_4_OS: 309.0805. Found: 309.0802.

4-((4-Chlorophenyl)thio)tetrazolo[1,5-*a*]quinoline (**3l**): White solid, mp: 174–177 °C. Yield: 55%. ^1^H NMR (400 MHz, CDCl_3_) δ: 8.61 (d, *J* = 8.2 Hz, 1H), 7.78 (dd, *J* = 15.2 and 7.6 Hz, 2H), 7.65 (d, *J* = 7.5 Hz, 1H), 7.60 (d, *J* = 8.3 Hz, 2H), 7.45 (d, *J* = 8.3 Hz, 2H), 7.36 (s, 1H). ^13^C NMR (100 MHz, CDCl_3_) δ: 146.5, 136.2, 135.8 (2C), 130.4, 130.3 (2C), 129.2, 129.0, 128.2, 127.9, 127.8, 124.4, 124.2, 116.7. HRMS *m*/*z*: [M + H]^+^ Calcd. for C_15_H_9_ClN_4_S: 313.0309. Found: 313.0300.

## 4. Conclusions

In summary, we have described here an efficient and environmentally friendly strategy to prepare 4-(arylselanyl)- and 4-(arylthiol)tetrazolo[1,5-*a*]quinoline starting from easily prepared and bench-stable 2-azidobenzaldehyde and 2-(arylchalcogenyl)acetonitriles. The reactions proceeded at a gentle heating of 80 °C for only 4 h, affording a total of 12 products **3a–l** in good to excellent yields (50–98%). Further studies are ongoing to better characterize the pharmacological potential antinociceptive and antidepressive-like of these new compounds.

## Figures and Tables

**Table 1:** Optimization of the reaction conditions to prepare **3a** ^a^.

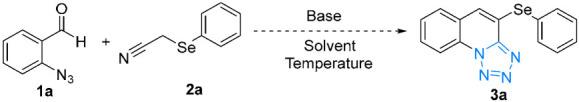					
Entry	Base	Solvent	Temperature (°C)	Time (h)	Yield (%) ^b^
1	K_2_CO_3_	DMSO	80	4	21
2	K_2_CO_3_	H_2_O	80	4	3
3	K_2_CO_3_	DMSO:H_2_O	80	4	98
4	K_2_CO_3_	glycerol:H_2_O	80	4	46
5	K_2_CO_3_	DMF:H_2_O	80	4	20
6	K_2_CO_3_	toluene:H_2_O	80	4	2
7	K_2_CO_3_	CH_3_CN:H_2_O	80	4	15
8	K_2_CO_3_	-	80	4	10
9	CsCO_3_	DMSO:H_2_O	80	4	92
10	KOH	DMSO:H_2_O	80	4	21
11	Et_3_N	DMSO:H_2_O	80	4	30
12	DBU	DMSO:H_2_O	80	4	72
13	K_2_CO_3_^c^	DMSO:H_2_O	80	4	10
14	-	DMSO:H_2_O	80	4	-
15	K_2_CO_3_	DMSO:H_2_O	60	4	30
16	K_2_CO_3_	DMSO:H_2_O	100	4	62
17	K_2_CO_3_	DMSO:H_2_O	80	2	55
18	K_2_CO_3_	DMSO:H_2_O	80	3	68
19	K_2_CO_3_	DMSO:H_2_O	25	4	50 ^d^

^a^ Reactions were performed using 2-azidobenzaldehyde **1a** (0.037 g, 0.25 mmol), 2-(arylselanyl)acetonitrile **2a** (0.049 g, 0.25 mmol) and base (0.007g, 20 mol%) in 1.0 mL of solvent under open atmosphere. ^b^ Yields are given for isolated product **3a**. ^c^ Used 10 mol% of K_2_CO_3_. ^d^ Obtained as a mixture of 1,2,3-triazolo[1,5-*a*]quinazoline **V** and **3a** (ratio **V**:**3a** = 2:8), as determined by ^1^H NMR and GC-MS analysis.

## Data Availability

The crystallographic information file (CIF) for the studied compound was deposited at the Cambridge Crystallographic Data Centre (CCDC) under identification number 2248272.

## Supplementary Materials

The following supporting information can be downloaded at: https://www.mdpi.com/article/10.3390/molecules28135036/s1, Figure S1–S31: Selected ^1^H, ^13^C, and ^77^Se NMR spectra of the prepared compounds; Figure S32: Thermal ellipsoid plot at the 50% probability level for the compound **3a**; Table S1: Crystal data and structure refinement for compound **3a**; Figures S33–S43: Selected HRMS spectra of the new compounds.

## Sample Availability

Samples of the compounds are available from the authors upon proper request.
