# Supplementary material for: Synthesis of 4-(Phenylchalcogenyl)tetrazolo[1,5-a]quinolines by Bicyclization of 2-Azidobenzaldehydes with Phenylchalcogenylacetonitrile
Source: Molecules. 2023 Jun 27;28(13):5036. doi: 10.3390/molecules28135036 (PMC10343424; doi:10.3390/molecules28135036)
Supplement: Supplementary file 1 [file molecules-28-05036-s001.zip › molecules-2452631-supplementary.pdf]

Supporting information for

Synthesis of 4-(phenylchalcogenyl)tetrazolo[1,5-*a*]quinolines by bicyclization of 2-azidobenzaldehydes with phenylchalcogenylacetonitrile.

Loana I. Monzon[1], Nicole C. M. Rocha[1], Gabriela T. Quadros[1], Pamela P. P. Nunes[1] Roberta Cargnelutti[2], Raquel G. Jacob[1], Eder J. Lenardão[1], Gelson Perin[1,*] and Daniela Hartwig[1,*]

Spectral information of the (4-(phenylchalcogenyl)tetrazolo[1,5-*a*]quinolines) products

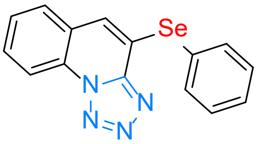

4-(Phenylselanyl)tetrazolo[1,5-*a*]quinoline (**3a**): Brown solid, mp: 171-174 °C. Yield: 98%. ^{1}H NMR (400 MHz, $CDCl_3$) d: 8.57 (d, J = 8.3 Hz, 1H), 7.77 (dd, J = 14.9 and 7.5 Hz, 3H), 7.67 (d, J = 7.8 Hz, 1H), 7.59 (t, J = 7.5 Hz, 1H), 7.49 (dt, J = 14.1 and 7.0 Hz, 3H), 7.37 (s, 1H). ^{13}C NMR (100 MHz, $CDCl_3$) δ: 147.1, 136.5 (2C), 130.9, 130.1 (2C), 130.0, 129.8, 129.2, 127.9, 127.7, 125.2, 124.6, 119.7, 116.6. ^{77}Se NMR (76 MHz, $CDCl_3$) δ: 397.49. HRMS *m/z*: $[M + H]^+$ Calcd. for $C_{15}H_{10}N_4Se$: 327.0143. Found: 327.0134.

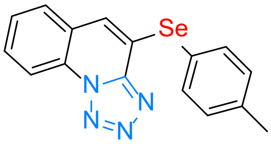

4-(*p*-Tolylselanyl)tetrazolo[1,5-*a*]quinoline (**3b**): Brown solid, mp: 123-125 °C. Yield: 60%. ^{1}H NMR (400 MHz, $CDCl_3$) δ: 8.56 (d, J = 8.3 Hz, 1H), 7.74 (t, J = 8.3 Hz, 1H), 7.66 (d, J = 8.0 Hz, 3H), 7.58 (t, J = 7.6 Hz, 1H), 7.31 (s, 1H), 7.27 (d, J = 7.9 Hz, 2H), 2.24 (s, 3H). ^{13}C NMR (100 MHz, $CDCl_3$) δ: 147.0, 140.2, 136.7 (2C), 131.0 (2C), 130.2, 129.8, 129.1, 127.9, 127.6, 124.6, 121.3, 120.4, 116.6, 21.3. ^{77}Se NMR (76 MHz, $CDCl_3$) δ: 353.31. HRMS *m/z*: $[M + H]^+$ Calcd. for $C_{16}H_{12}N_4Se$: 341.0300. Found: 341.0290.

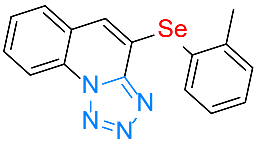

4-(*o*-Tolylselanyl)tetrazolo[1,5-*a*]quinoline (**3c**): White solid, mp: 167–169 °C. Yield: 63%. ^{1}H NMR (400 MHz, $CDCl_3$) δ: 8.56 (d, J = 8.3 Hz, 1H), 7.77-7.72 (m, 2H), 7.65 (d, J = 7.4 Hz, 1H), 7.58 (t, J = 7.5 Hz, 1H), 7.43 (d, J = 4.2 Hz, 2H), 7.28-7.24 (m, 1H), 7.14 (s, 1H), 2.51 (s, 3H). ^{13}C NMR (100 MHz, $CDCl_3$) δ: 147.0, 142.9, 137.9, 130.9, 130.5, 129.8, 129.1, 129.0, 127.9, 127.6, 127.5, 125.7, 124.6, 119.3, 116.5, 22.7. ^{77}Se NMR (76 MHz, $CDCl_3$) δ: 354.05. HRMS *m/z*: $[M + H]^+$ Calcd. for $C_{16}H_{12}N_4Se$: 341.0300. Found: 341.0287.

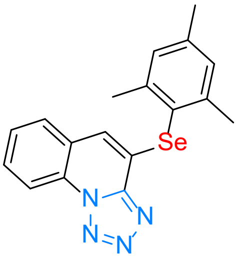

4-(Mesitylselanyl)tetrazolo[1,5-*a*]quinoline (**3d**): White solid, mp: 184–186 °C. Yield: 45%. ^{1}H NMR (400 MHz, $CDCl_3$) δ: 8.49 (d, J = 8.1 Hz, 1H), 7.64 (t, J = 7.3 Hz, 1H), 7.55 (d, J = 7.5 Hz, 1H), 7.48 (t, J = 7.2 Hz, 1H), 7.03 (s, 2H), 6.83 (s, 1H), 2.41 (s, 6H), 2.31 (s, 3H). ^{13}C NMR (100 MHz, $CDCl_3$) δ: 147.09, 144.1 (2C), 140.61, 129.4 (2C), 129.38, 128.89, 127.80, 127.44, 127.08, 124.89, 122.94, 119.82, 116.61, 23.9 (2C), 21.15. ^{77}Se NMR (76 MHz, $CDCl_3$) δ: 280.22. HRMS *m/z*: $[M + H]^+$ Calcd. for $C_{18}H_{16}N_4Se$: 369.0613. Found: 369.0608.

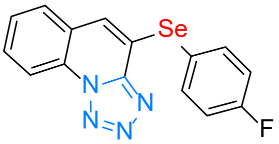

4-((4-Fluorophenyl)selanyl)tetrazolo[1,5-*a*]quinoline (**3e**): Yellow solid, mp: 163-167 °C. Yield: 80%. ^{1}H NMR (400 MHz, $CDCl_3$) δ: 8.55 (d, J = 8.3 Hz, 1H), 7.79-7.72 (m, 3H), 7.66 (d, J = 7.9 Hz, 1H), 7.58 (t, J = 8.1 Hz, 1H), 7.52-7.44 (m, 2H), 7.36 (s, 1H). ^{13}C NMR (100 MHz, $CDCl_3$) δ: 163.8 (d, J = 251.8 Hz), 146.3, 137.4 (d, J = 8.6 Hz, 2C), 130.1, 129.0, 128.1, 127.8, 127.8, 125.4, 124.3, 124.0 (d, J = 3.4 Hz), 117.4 (d, J = 22.1 Hz, 2C), 116.7. ^{77}Se NMR (76 MHz, $CDCl_3$) δ: 388.82. HRMS *m/z*: $[M + H]^+$ Calcd. for $C_{15}H_9FN_4Se$: 345.0049. Found: 345.0052.

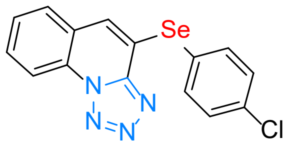

4-((4-Chlorophenyl)selanyl)tetrazolo[1,5-*a*]quinoline (**3f**): White solid, mp: 146–149 °C. Yield: 52%. ^{1}H NMR (400 MHz, $CDCl_3$) δ: 8.58 (d, *J* = 8.3 Hz, 1H), 7.79 (t, *J* = 8.3 Hz, 1H), 7.71 (d, *J* = 8.5 Hz, 3H), 7.63 (t, *J* = 7.6 Hz, 1H), 7.45 (s, 1H), 7.41 (d, *J* = 8.4 Hz, 2H). ^{13}C NMR (100 MHz, $CDCl_3$) δ: 147.1, 137.6 (2C), 136.2, 131.6, 130.4, 130.3 (2C), 129.4, 128.1, 127.9, 124.4, 123.6, 118.8, 116.7. ^{77}Se NMR (76 MHz, $CDCl_3$) δ: 391.28. HRMS *m/z*: $[M + H]^+$ Calcd. for $C_{15}H_9ClN_4Se$: 360.9754. Found: 360.9735.

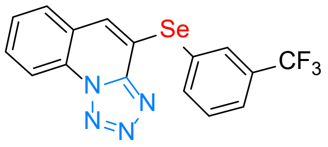

4-((3-(Trifluoromethyl)phenyl)selanyl)tetrazolo[1,5-*a*]quinoline (**3g**): Yellow solid, mp: 145-149 °C. Yield: 50%. ^{1}H NMR (400 MHz, $CDCl_3$) δ: 8.62 (d, *J* = 8.3 Hz, 1H), 8.03 (s, 1H), 7.97 (dd, *J* = 7.8 Hz, 1H), 7.84 (td, *J* = 7.3 Hz, 1H), 7.77 (d, *J* = 8.0 Hz, 1H), 7.73-7.71 (m, 1H), 7.66 (td, *J* = 8.6 Hz, 1H), 7.58 (s, 1H), 7.56 (d, *J* = 8.1 Hz, 1H). ^{13}C NMR (100 MHz, $CDCl_3$) δ: 147.3, 139.2, 133.1, 132.3 (q, *J* = 3.8 Hz), 132.2 (q, *J* = 32.8 Hz), 130.8, 130.4, 128.7, 128.4 (q, *J* = 266.0 Hz), 128.2, 128.1, 126.3 (q, *J* = 3.8 Hz), 124.7, 122.0, 117.6, 116.8. ^{77}Se NMR (76 MHz, $CDCl_3$) δ: 400.57. HRMS *m/z*: $[M + H]^+$ Calcd. for $C_{16}H_9F_3N_4Se$: 395.0017. Found: 395.0017.

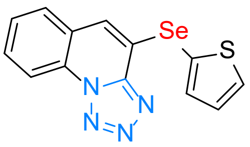

4-(Thiophen-2-ylselanyl)tetrazolo[1,5-*a*]quinoline (**3h**): White solid, mp: 160-163 °C. Yield: 70%. ^{1}H NMR (400 MHz, $CDCl_3$) δ: 8.54 (d, *J* = 8.3 Hz, 1H), 7.75 (td, *J* = 8.4 and 1.3 Hz, 1H), 7.70-7.66 (m, 2H), 7.59 (td, *J* = 8.2 and 1.2 Hz, 1H), 7.55 (dd, *J* = 3.5 and 1.2 Hz, 1H), 7.31 (s, 1H), 7.23-7.21 (m, 1H). ^{13}C NMR (100 MHz, $CDCl_3$) δ: 146.5, 139.4, 134.1, 130.10, 130.09, 129.1, 129.0, 127.9, 127.8, 124,5, 120.4, 117.9, 116.5. HRMS *m/z*: $[M + H]^+$ Calcd. for $C_{13}H_8N_4SSe$: 332.9708. Found: 332.9701.

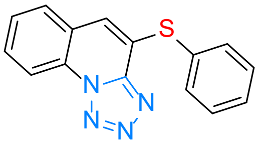

4-(Phenylthio)tetrazolo[1,5-*a*]quinoline (**3i**): Brown solid, mp: 175-178 °C. Yield: 62%. ^{1}H NMR (400 MHz, $CDCl_3$) δ: 8.57 (d, *J* = 8.3 Hz, 1H), 7.75 (t, *J* = 8.3 Hz, 1H), 7.70-7.66 (m, 3H), 7.60 (t, *J* = 7.6 Hz, 1H), 7.51-7.49 (m, 3H), 7.24 (s, 1H). ^{13}C NMR (100 MHz, $CDCl_3$) δ: 146.4, 134.9 (2C), 130.1 (2C), 129.9, 129.9, 128.9, 128.8, 128.0, 127.8 (2C), 125.4, 124.3, 116.6. HRMS *m/z*: $[M + H]^+$ Calcd. for $C_{15}H_{10}N_4S$: 279.0699. Found: 279.0692.

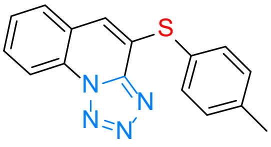

4-(*p*-Tolylthio)tetrazolo[1,5-*a*]quinoline (**3j**): Brown solid, mp: 122-126 °C. Yield: 60%. ^{1}H NMR (400 MHz, DMSO-d_6) δ: 8.67 (d, *J* = 8.3 Hz, 1H), 8.19 (d, *J* = 7.9 Hz, 1H), 8.02 (t, *J* = 7.6 Hz, 1H), 7.87-7.83 (m, 2H), 7.66 (d, *J* = 8.0 Hz, 2H), 7.46 (d, *J* = 7.9 Hz, 2H), 3.47 (s, 3H). ^{13}C NMR (100 MHz, DMSO) δ: 146.3, 139.2, 133.4 (2C), 130.8 (2C), 130.7, 130.1, 128.8, 128.6, 128.3, 125.8, 124.1, 122.8, 116.1, 20.8. HRMS *m/z*: $[M + H]^+$ Calcd. for $C_{16}H_{12}N_4S$: 293.0855. Found: 293.0851.

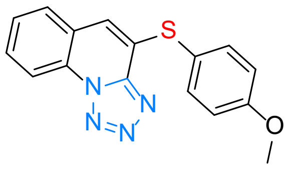

4-((4-Methoxyphenyl)thio)tetrazolo[1,5-*a*]quinoline (**3k**): White solid, mp: 157–159 °C. Yield: 55%. ^{1}H NMR (400 MHz, $CDCl_3$) δ: 8.55 (d, *J* = 8.3 Hz, 1H), 7.71 (t, *J* = 7.7 Hz, 1H), 7.67-7.56 (m, 4H), 7.10-7.01 (m, 3H), 3.90 (s, 3H). ^{13}C NMR (100 MHz, $CDCl_3$) δ: 161.3, 146.1, 137.4 (2C), 129.5, 128.6, 127.9, 127.6, 127.1, 125.9, 124.4, 118.3, 116.5, 115.7 (2C), 55.5. HRMS *m/z*: $[M + H]^+$ Calcd. for $C_{16}H_{12}N_4OS$: 309.0805. Found: 309.0802.

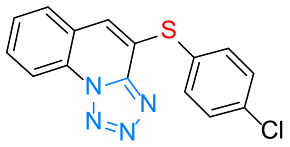

4-((4-Chlorophenyl)thio)tetrazolo[1,5-*a*]quinoline (**3l**): White solid, mp: 174–177 °C. Yield: 55%. ^{1}H NMR (400 MHz, $CDCl_3$) δ: 8.61 (d, *J* = 8.2 Hz, 1H), 7.78 (dd, *J* = 15.2 and 7.6 Hz, 2H), 7.65 (d, *J* = 7.5 Hz, 1H), 7.60 (d, *J* = 8.3 Hz, 2H), 7.45 (d, *J* = 8.3 Hz, 2H), 7.36 (s, 1H). ^{13}C NMR (100 MHz, $CDCl_3$) δ: 146.5, 136.2, 135.8 (2C), 130.4, 130.3 (2C), 129.2, 129.0, 128.2, 127.9, 127.8, 124.4, 124.2, 116.7. HRMS *m/z*: $[M + H]^+$ Calcd. for $C_{15}H_9ClN_4S$: 313.0309. Found: 313.0300.

Selected NMR spectra

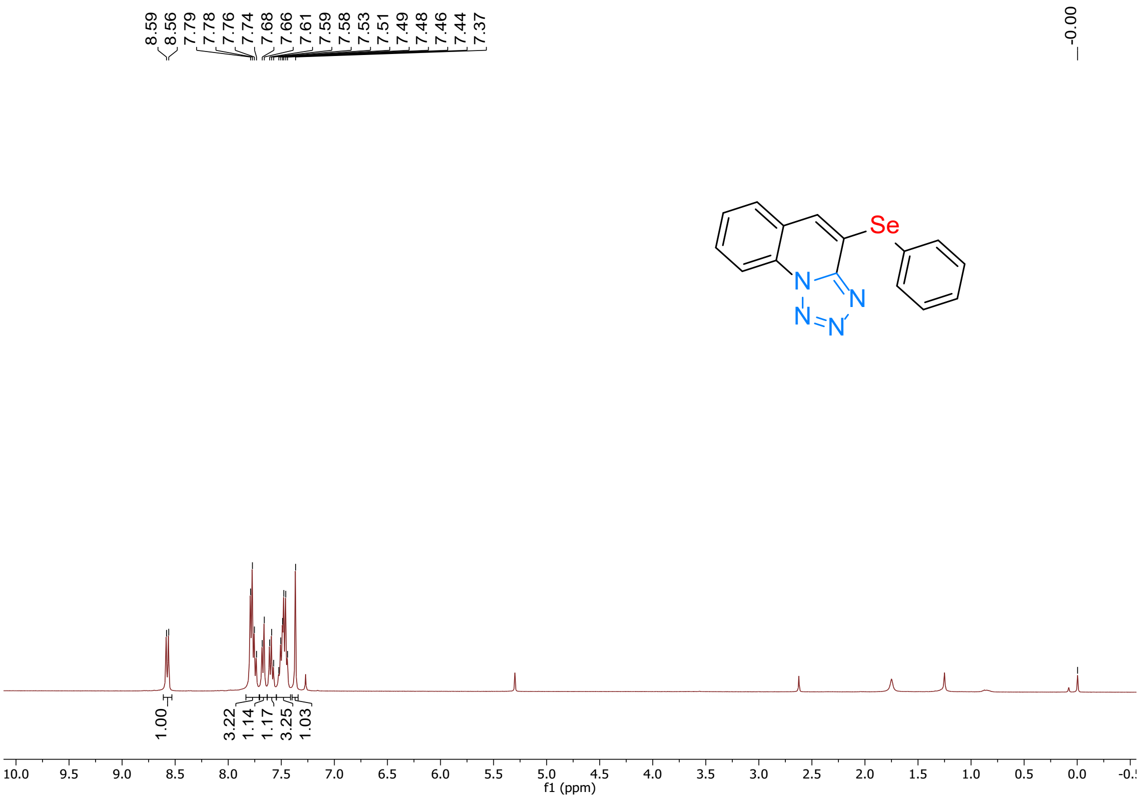

Figure S1. ^{1}H NMR (400 MHz) spectrum for compound **3a** in $CDCl_3$.

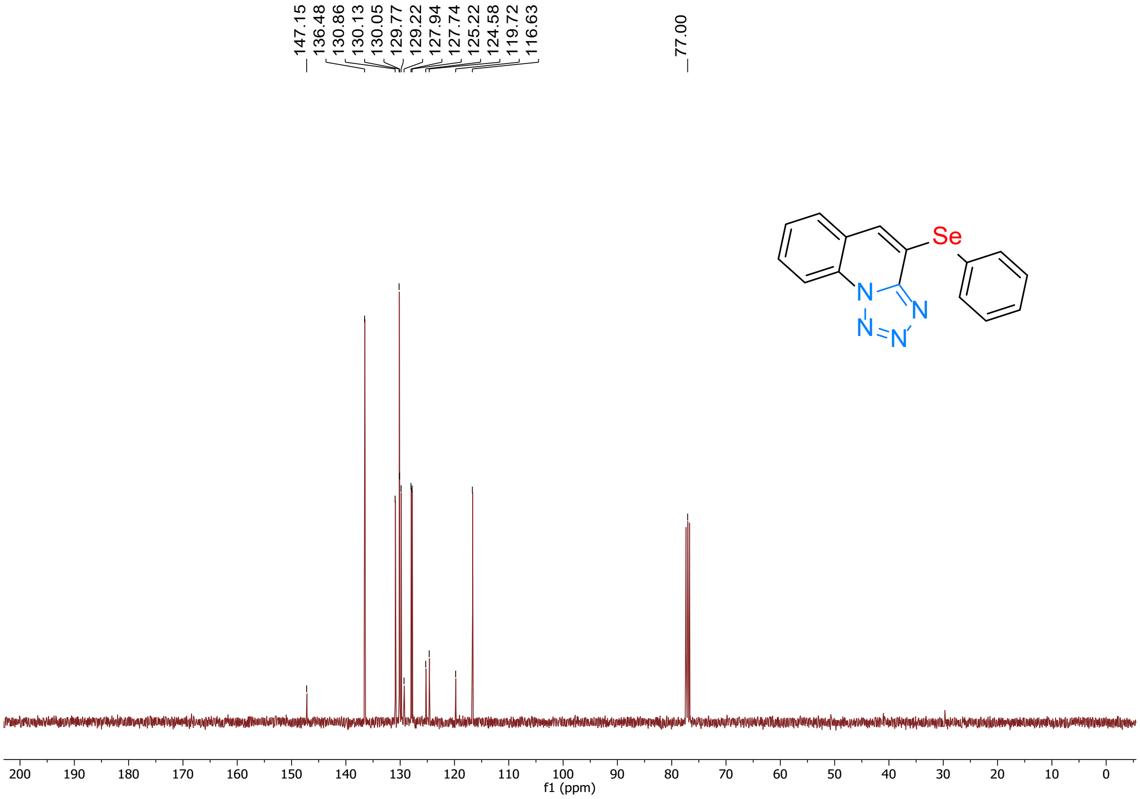

Figure S2. ^{13}C NMR (100 MHz) spectrum for compound **3a** in $CDCl_3$.

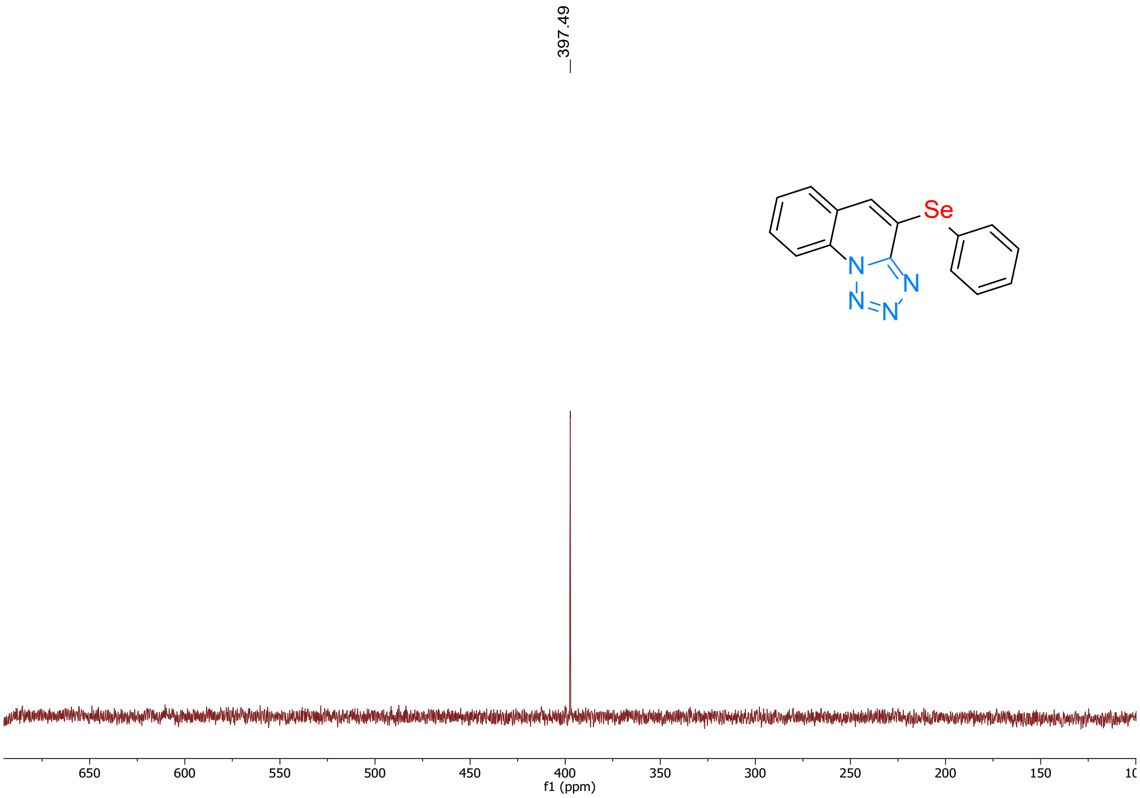

Figure S3. ^{77}Se NMR (76 MHz) spectrum for compound **3a** in CDCl3.

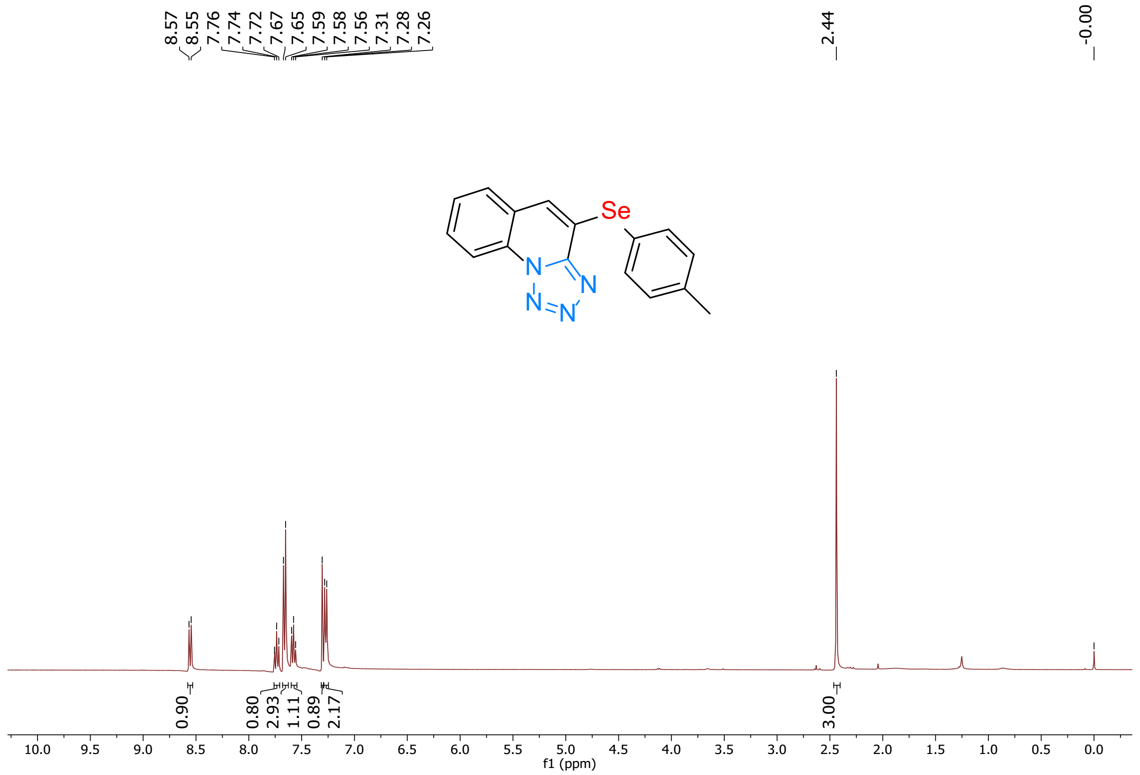

Figure S4. ^{1}H NMR (400 MHz) spectrum for compound **3b** in CDCl3.

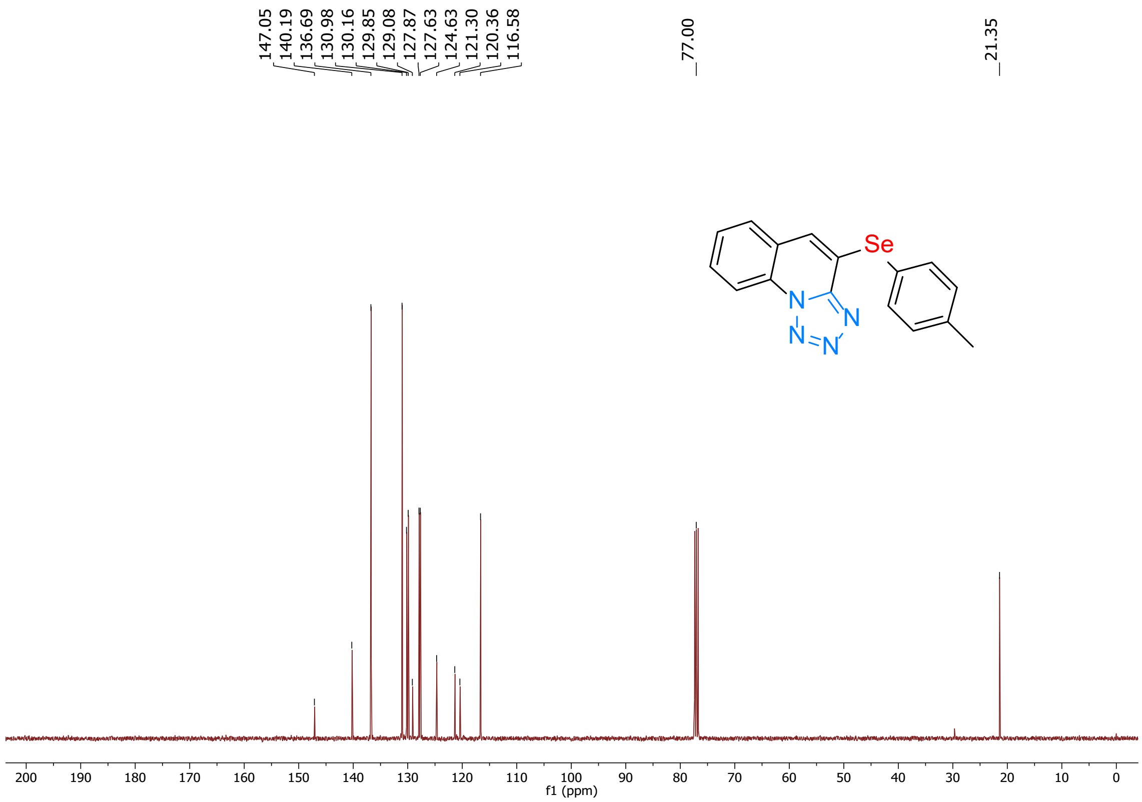

Figure S5. ^{13}C NMR (100 MHz) spectrum for compound **3b** in $CDCl_3$.

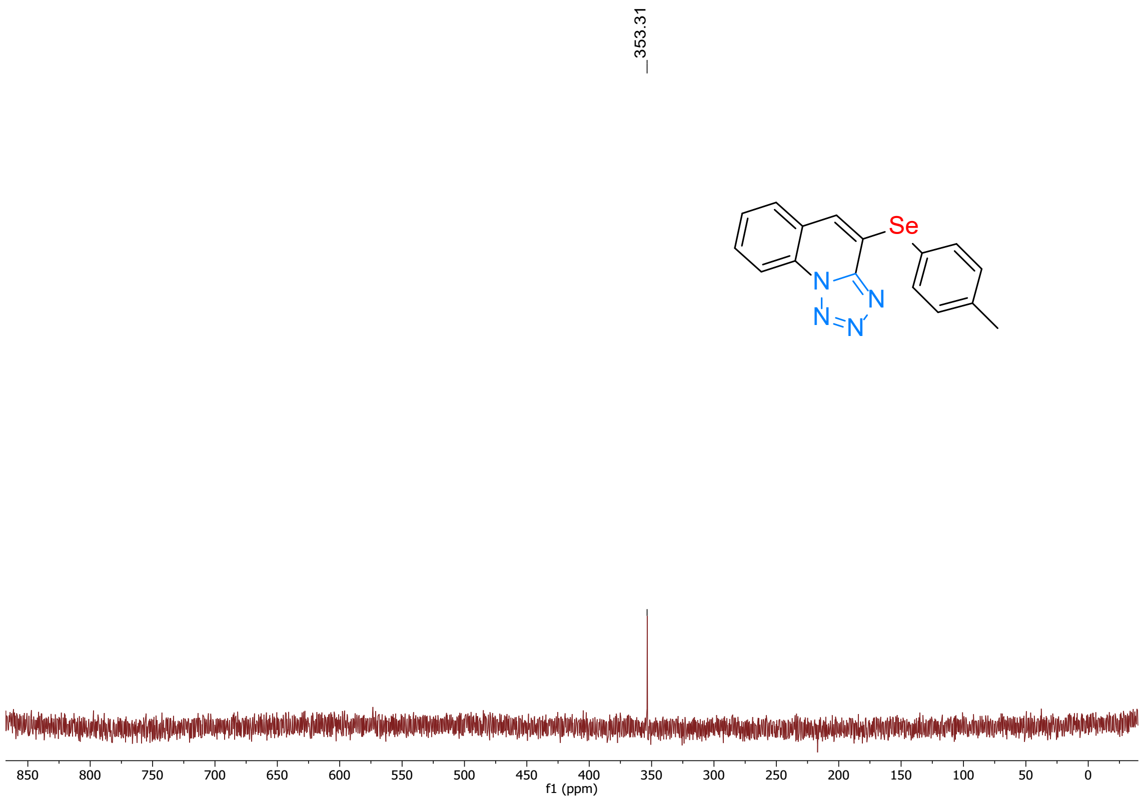

Figure S6. ^{77}Se NMR (76 MHz) spectrum for compound **3b** in $CDCl_3$.

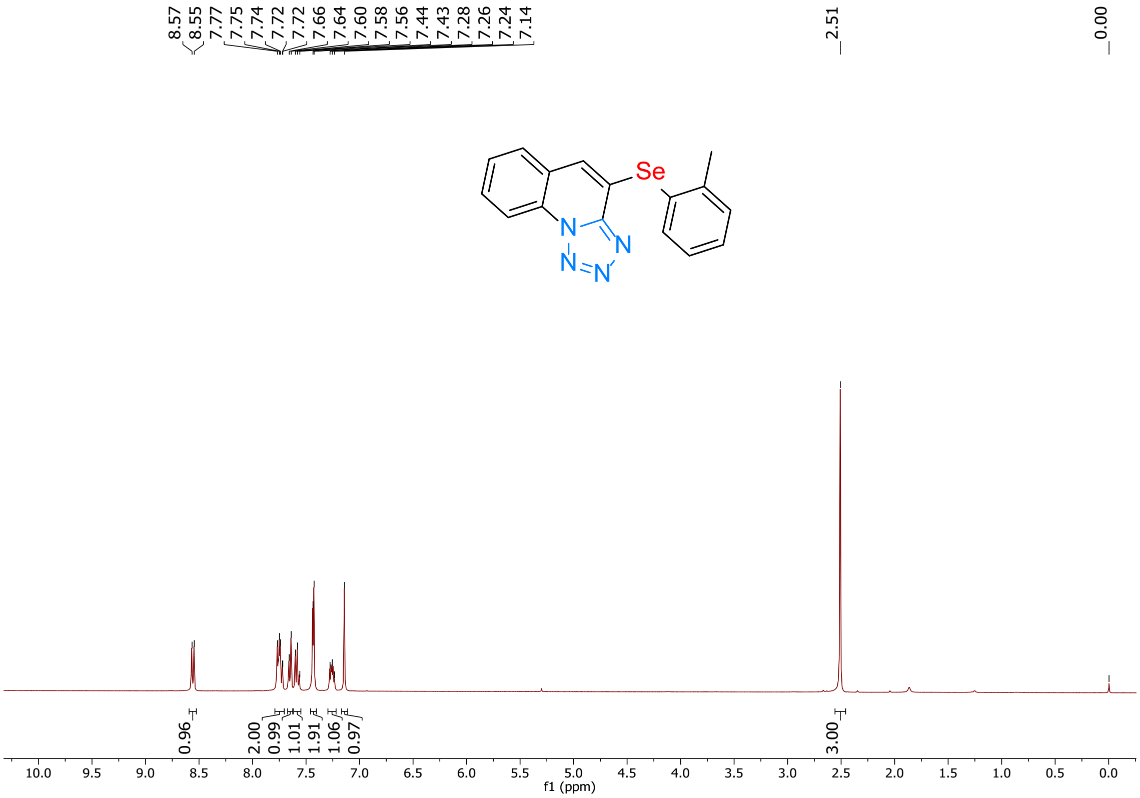

Figure S7. ^{1}H NMR (400 MHz) spectrum for compound **3c** in $CDCl_3$.

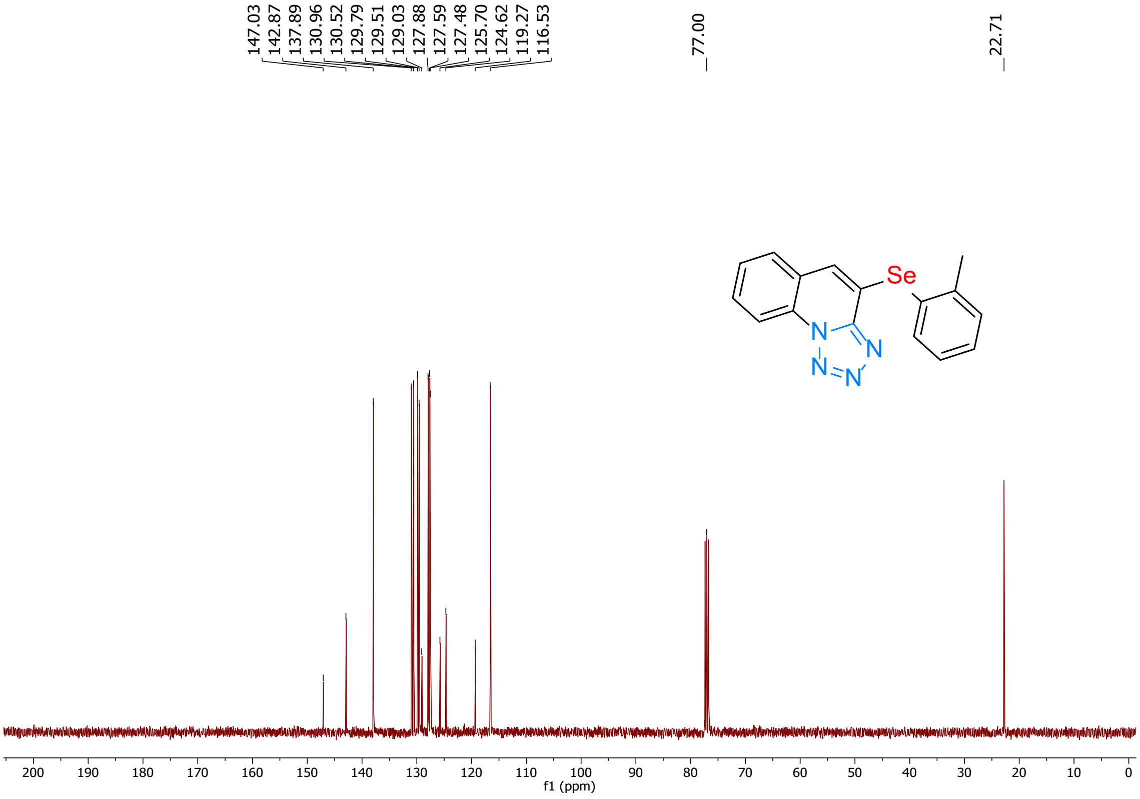

Figure S8. ^{13}C NMR (100 MHz) spectrum for compound **3c** in $CDCl_3$.

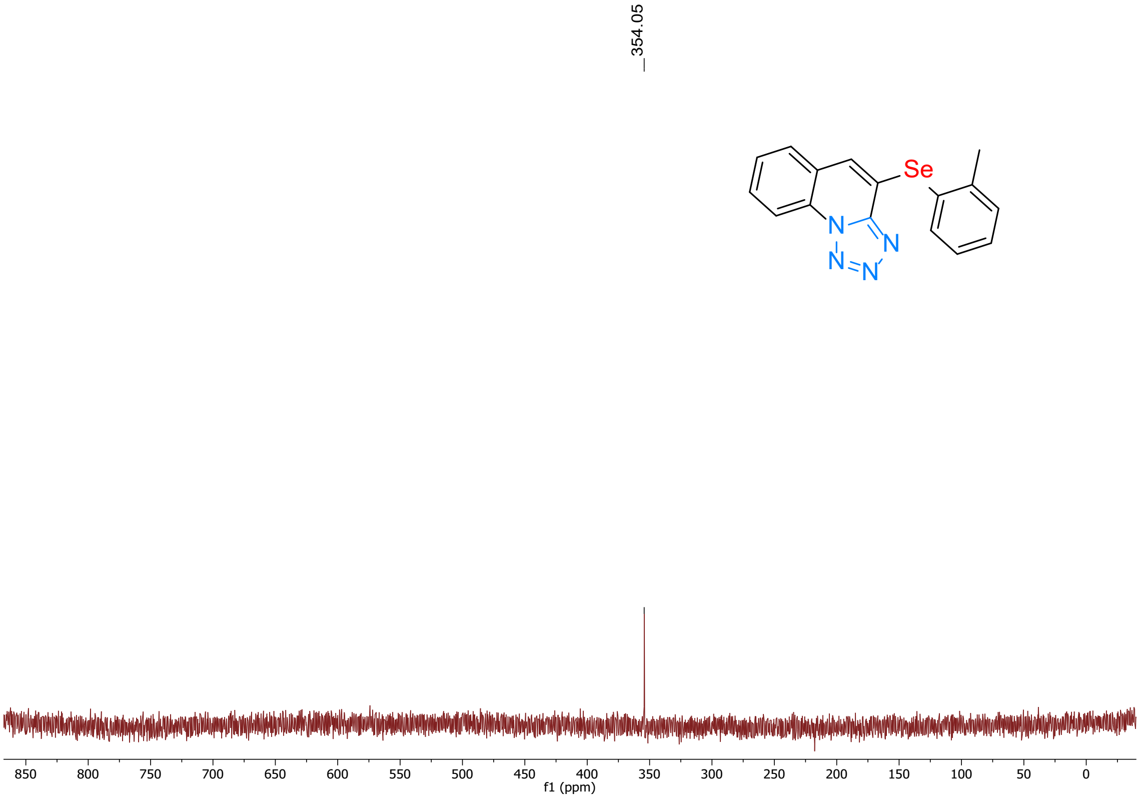

Figure S9. ^{77}Se NMR (76 MHz) spectrum for compound **3c** in $CDCl_3$.

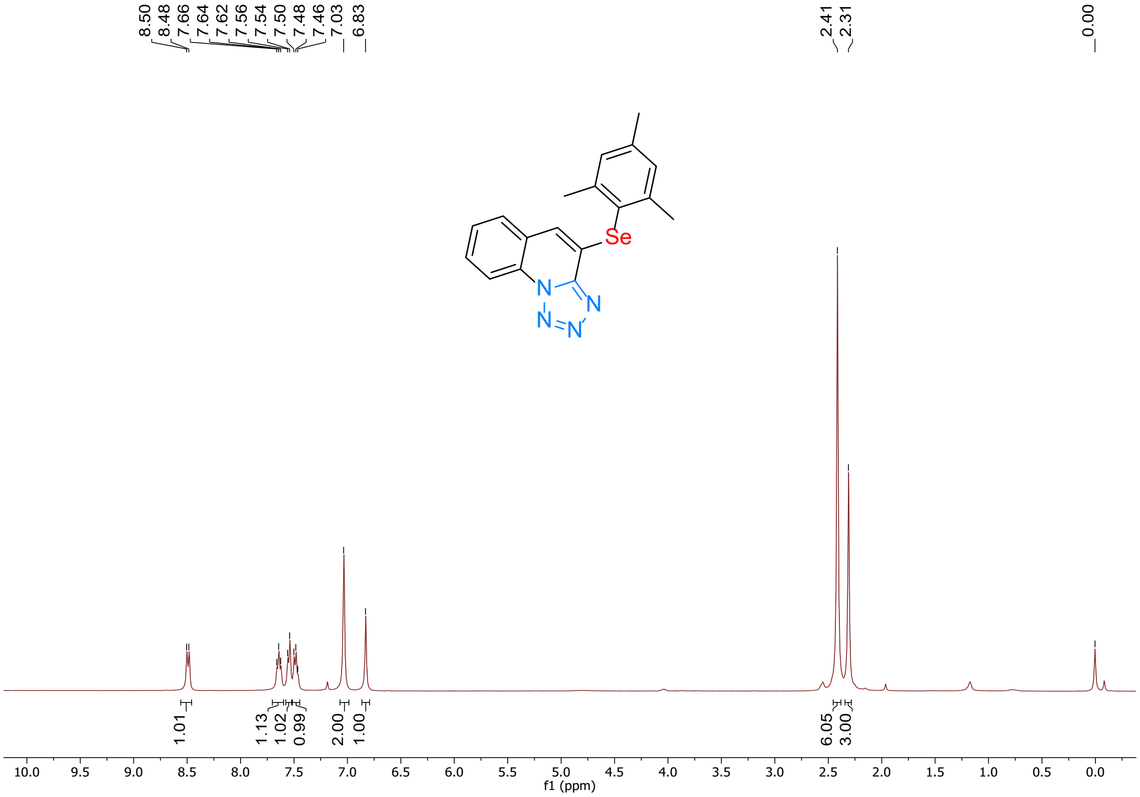

Figure S10. ^{1}H NMR (400 MHz) spectrum for compound **3d** in $CDCl_3$.

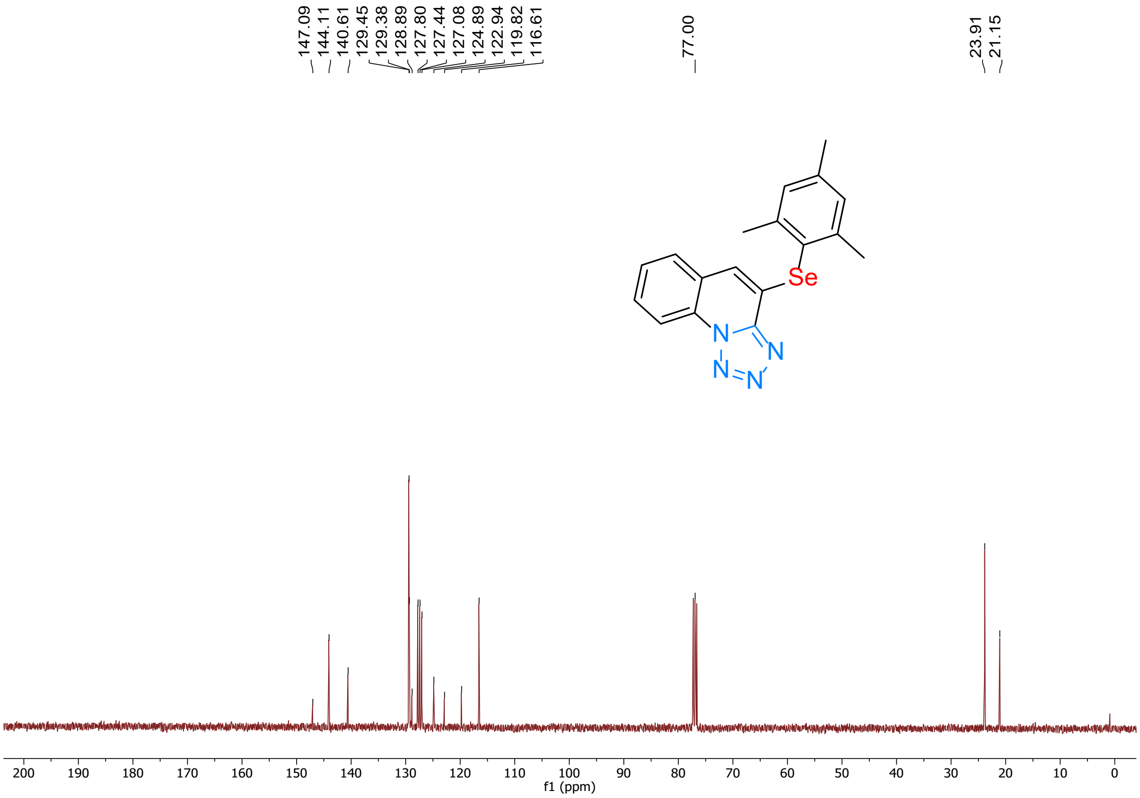

Figure S11. ^{13}C NMR (100 MHz) spectrum for compound **3d** in $CDCl_3$.

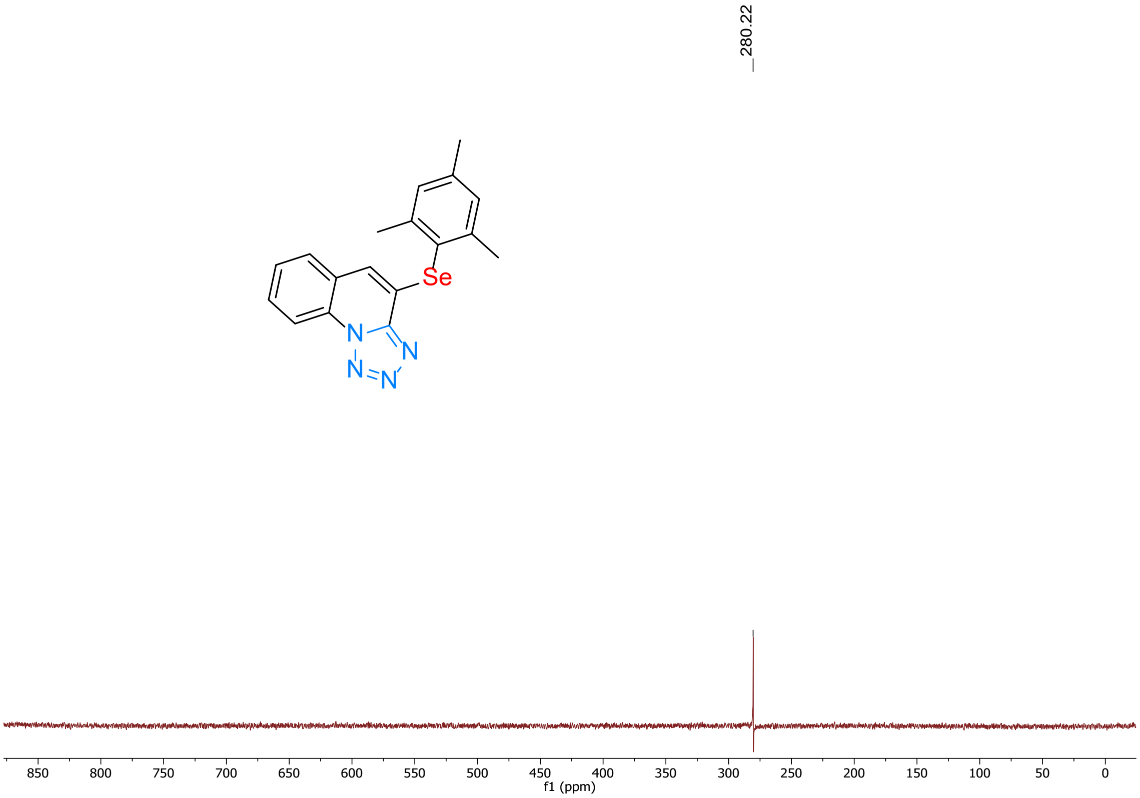

Figure S12. ^{77}Se NMR (76 MHz) spectrum for compound **3d** in $CDCl_3$.

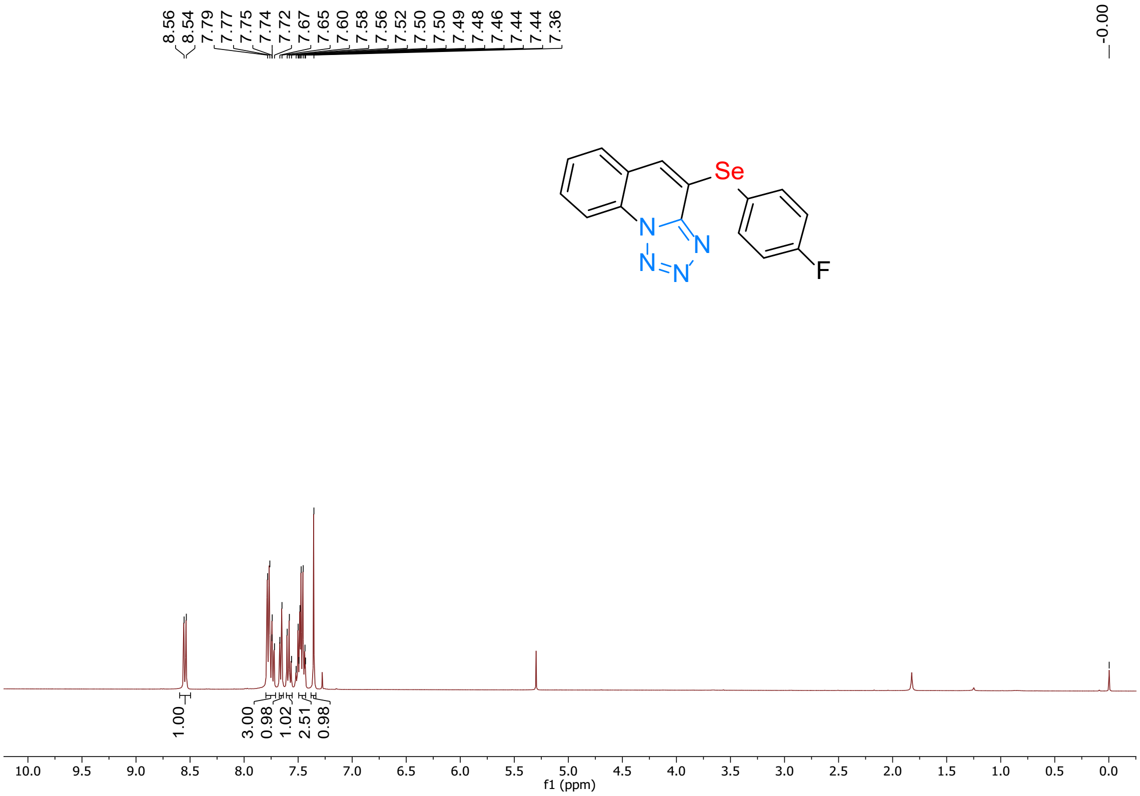

Figure S13. ^{1}H NMR (400 MHz) spectrum for compound **3e** in $CDCl_3$.

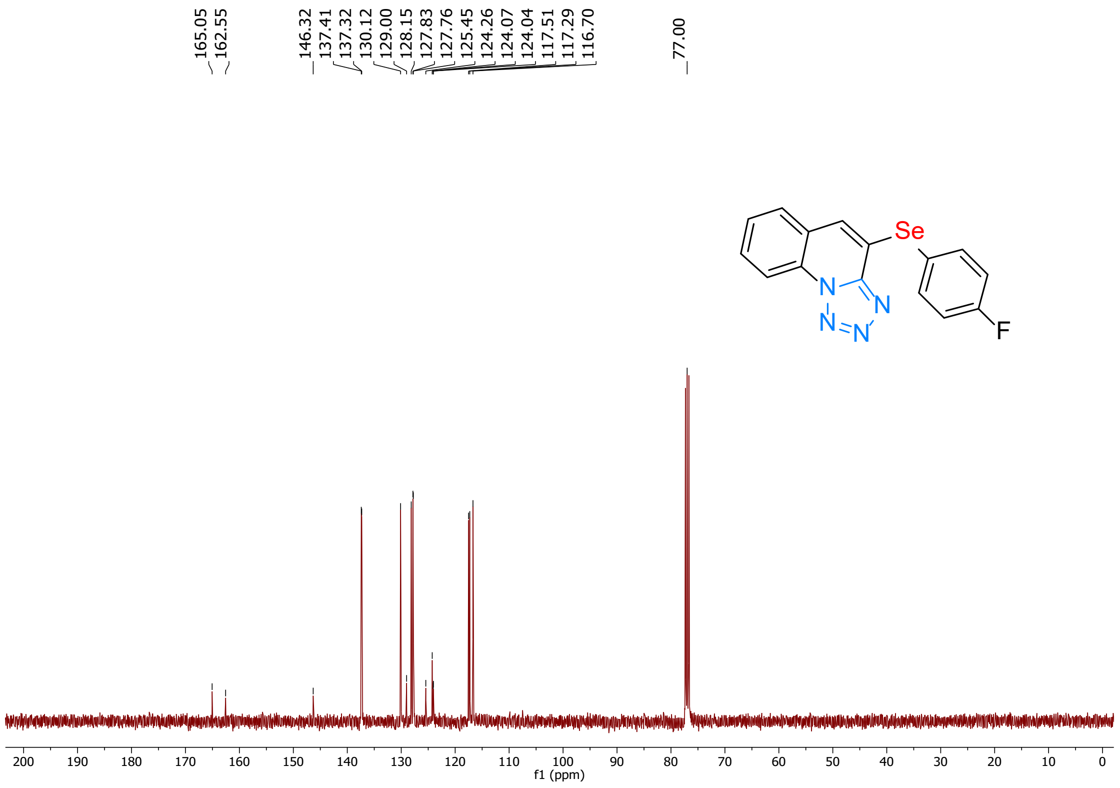

Figure S14. ^{13}C NMR (100 MHz) spectrum for compound **3e** in $CDCl_3$.

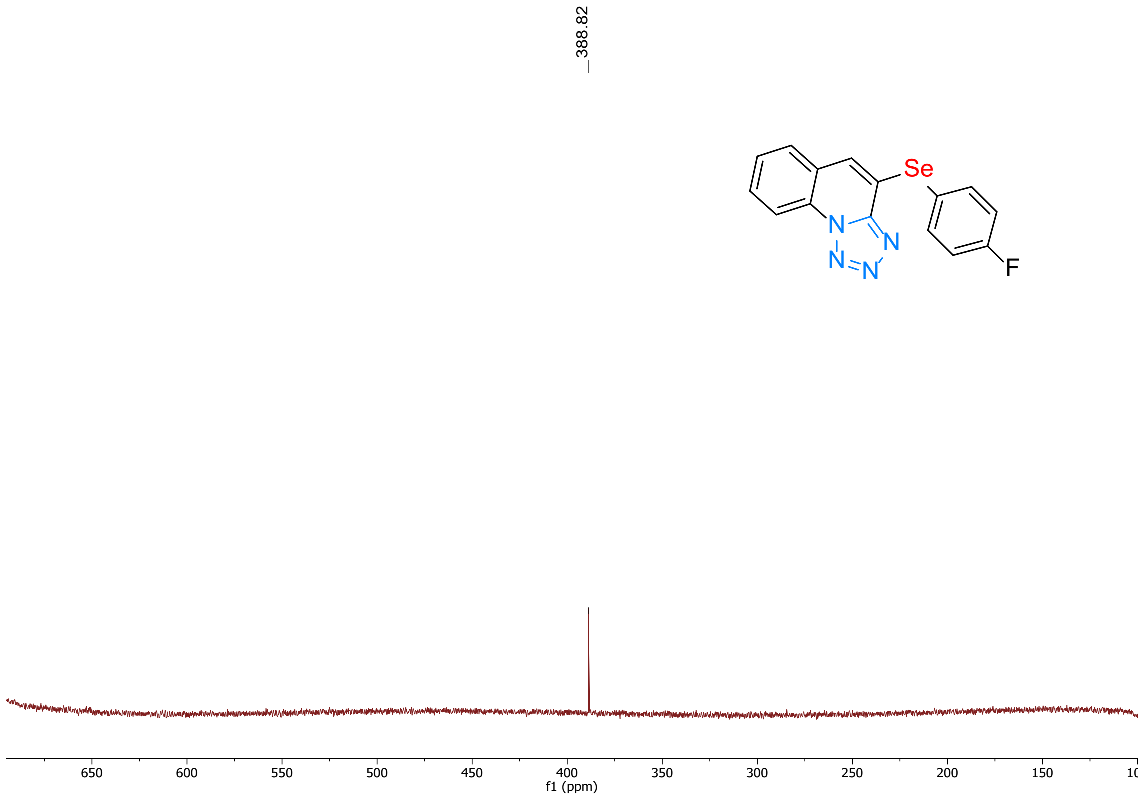

Figure S15. ^{77}Se NMR (76 MHz) spectrum for compound **3e** in $CDCl_3$.

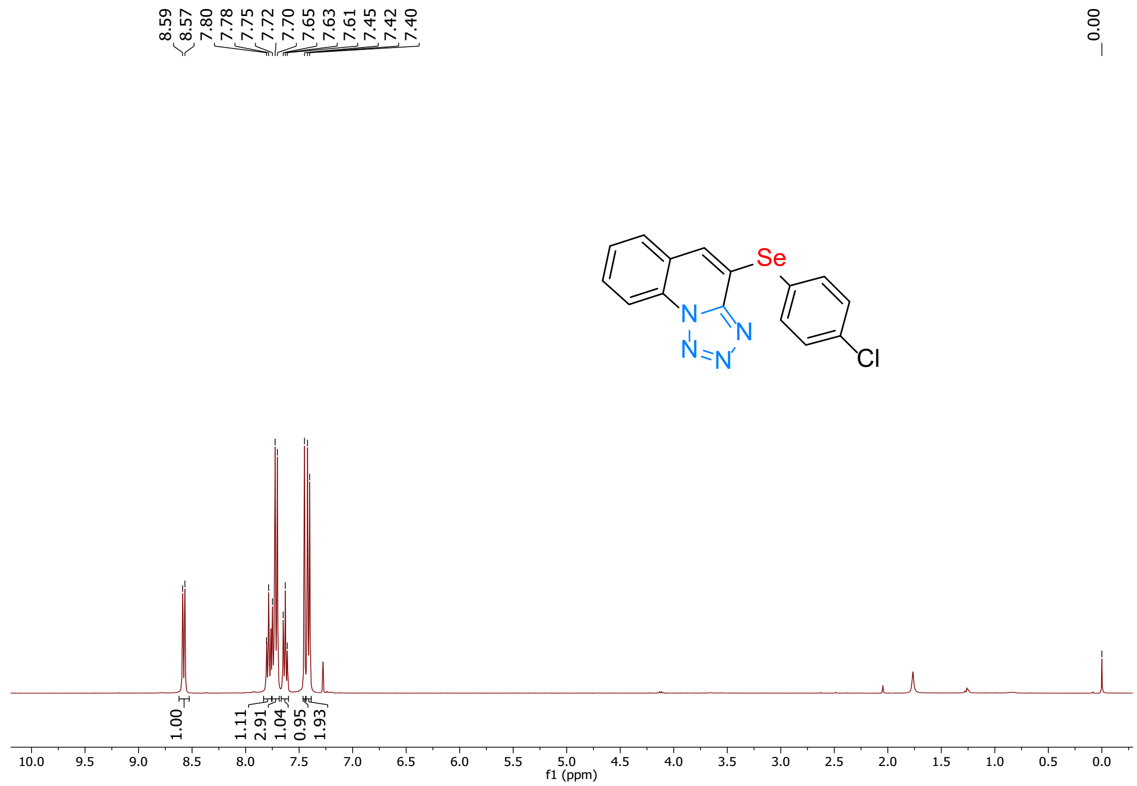

Figure S16. ^{1}H NMR (400 MHz) spectrum for compound **3f** in $CDCl_3$.

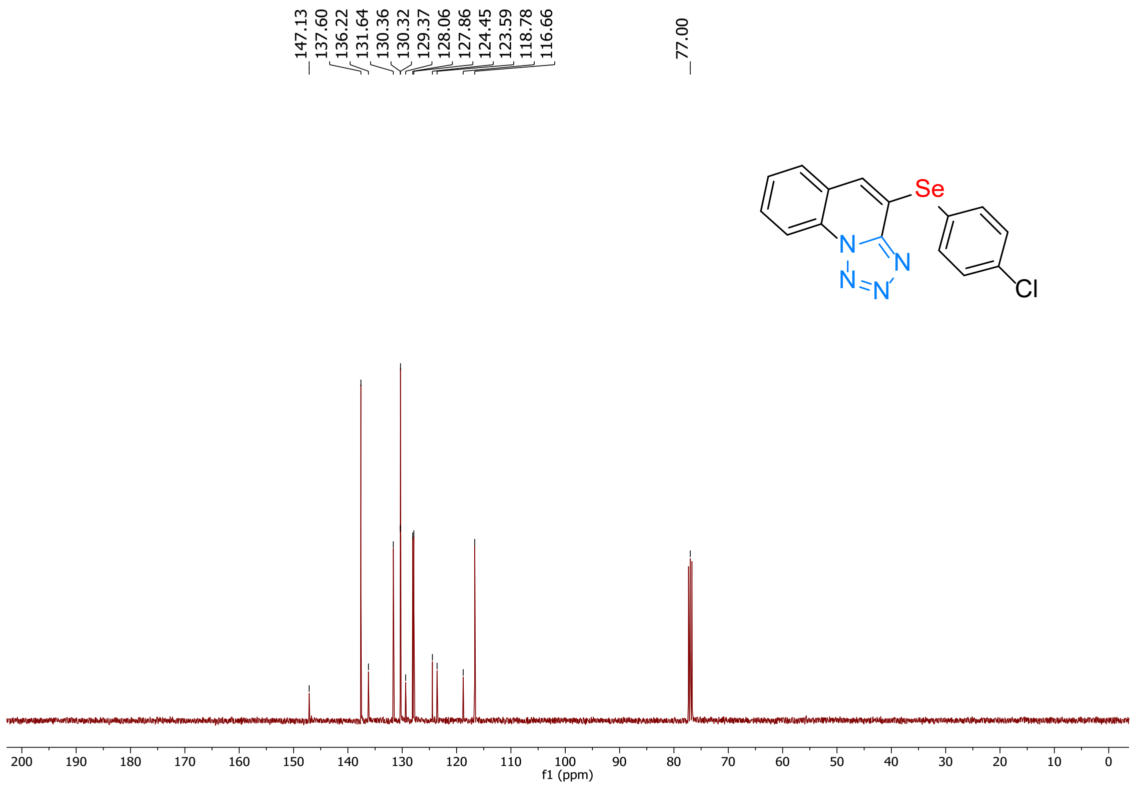

Figure S17. ^{13}C NMR (100 MHz) spectrum for compound **3f** in $CDCl_3$.

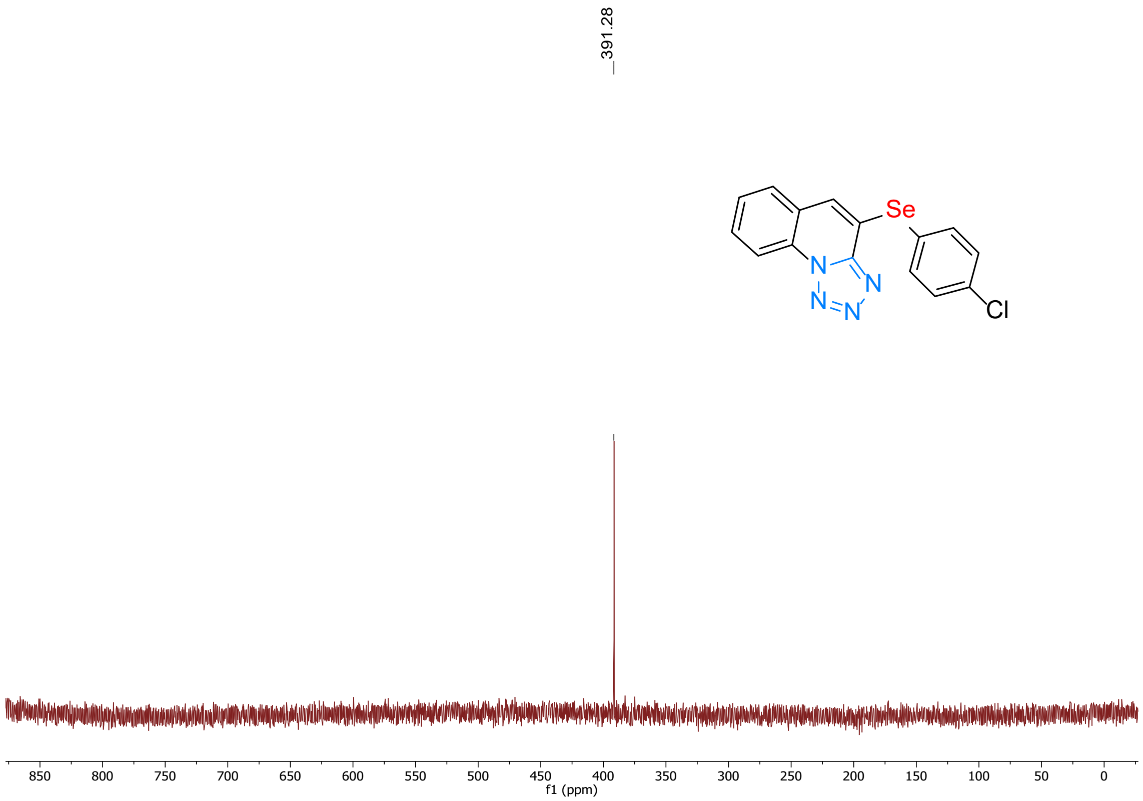

Figure S18. ^{77}Se NMR (76 MHz) spectrum for compound **3f** in $CDCl_3$.

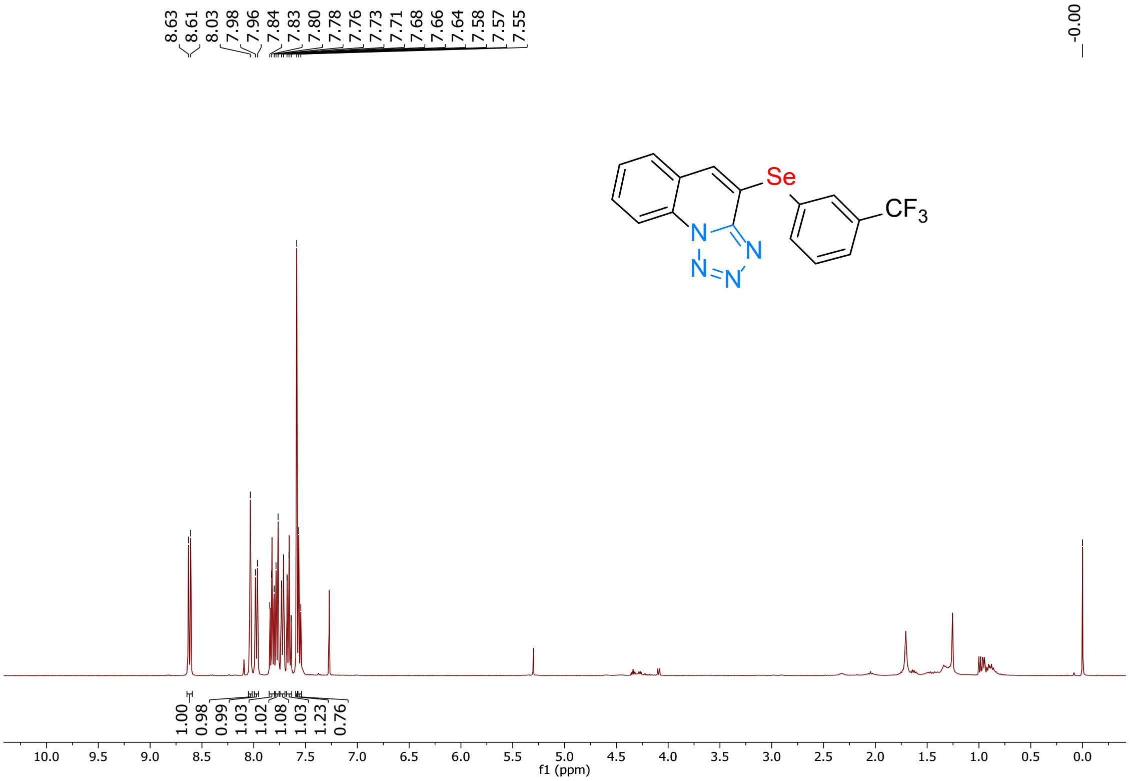

Figure S19. ^{1}H NMR (400 MHz) spectrum for compound **3g** in $CDCl_3$.

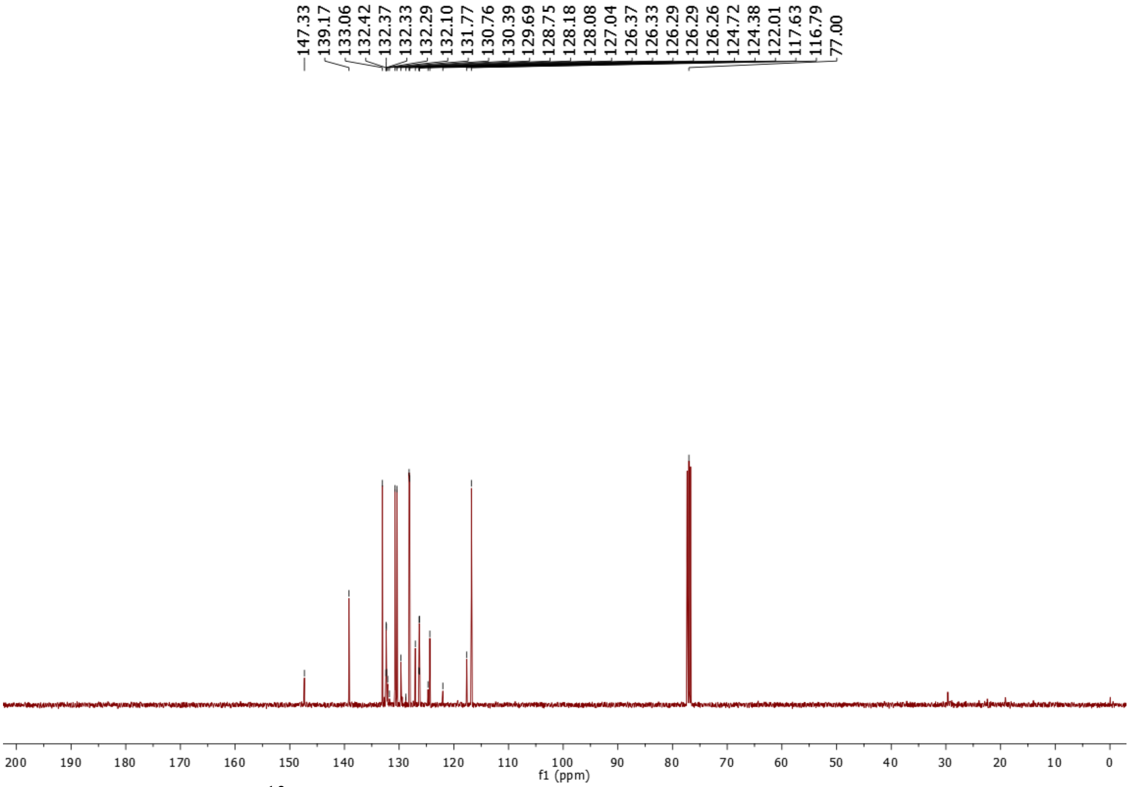

Figure S20. ^{13}C NMR (100 MHz) spectrum for compound **3g** in $CDCl_3$.

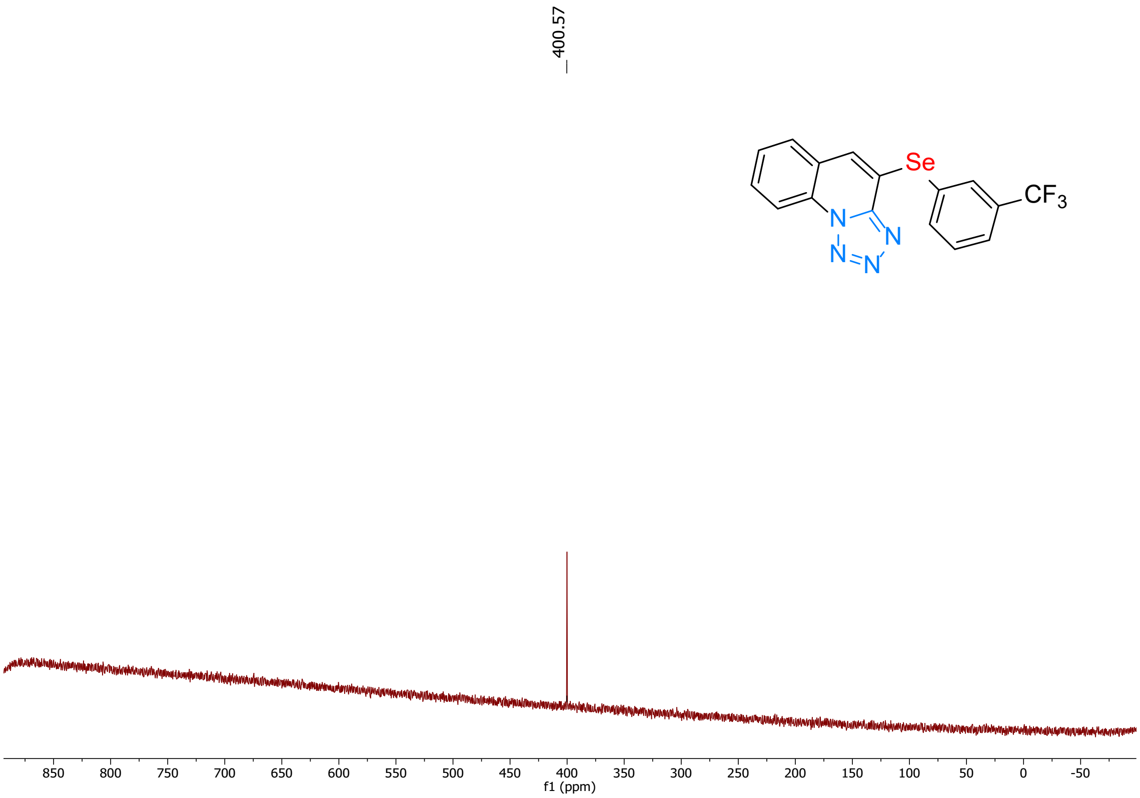

Figure S21. ^{77}Se NMR (76 MHz) spectrum for compound **3g** in $CDCl_3$.

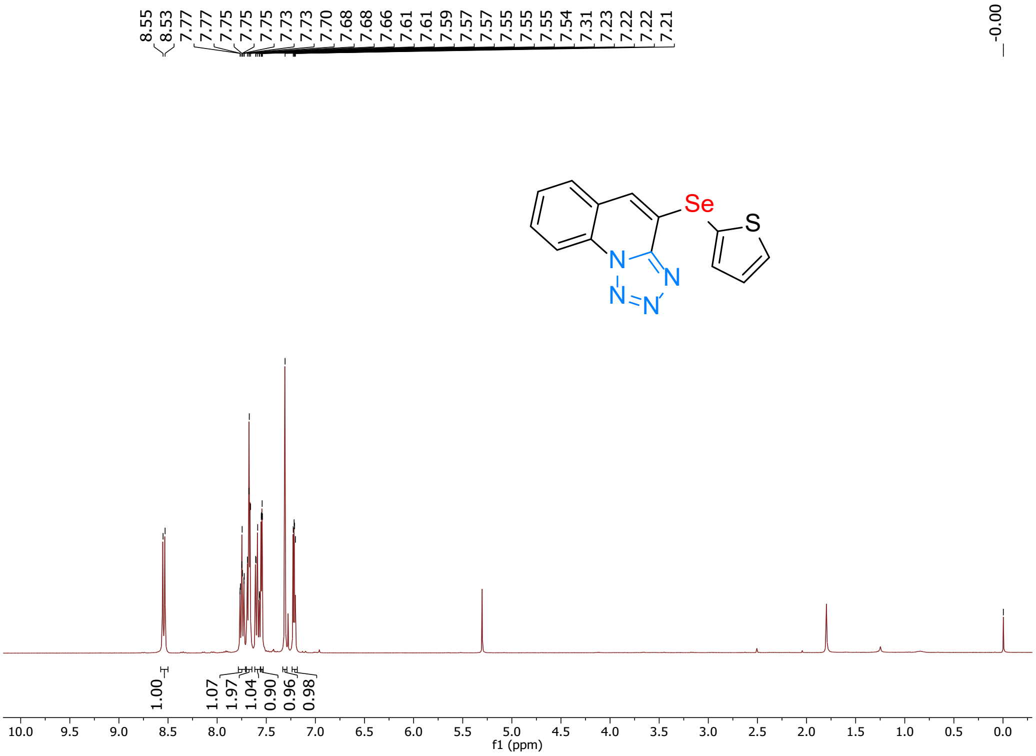

Figure S22. ^{1}H NMR (400 MHz) spectrum for compound **3h** in $CDCl_3$.

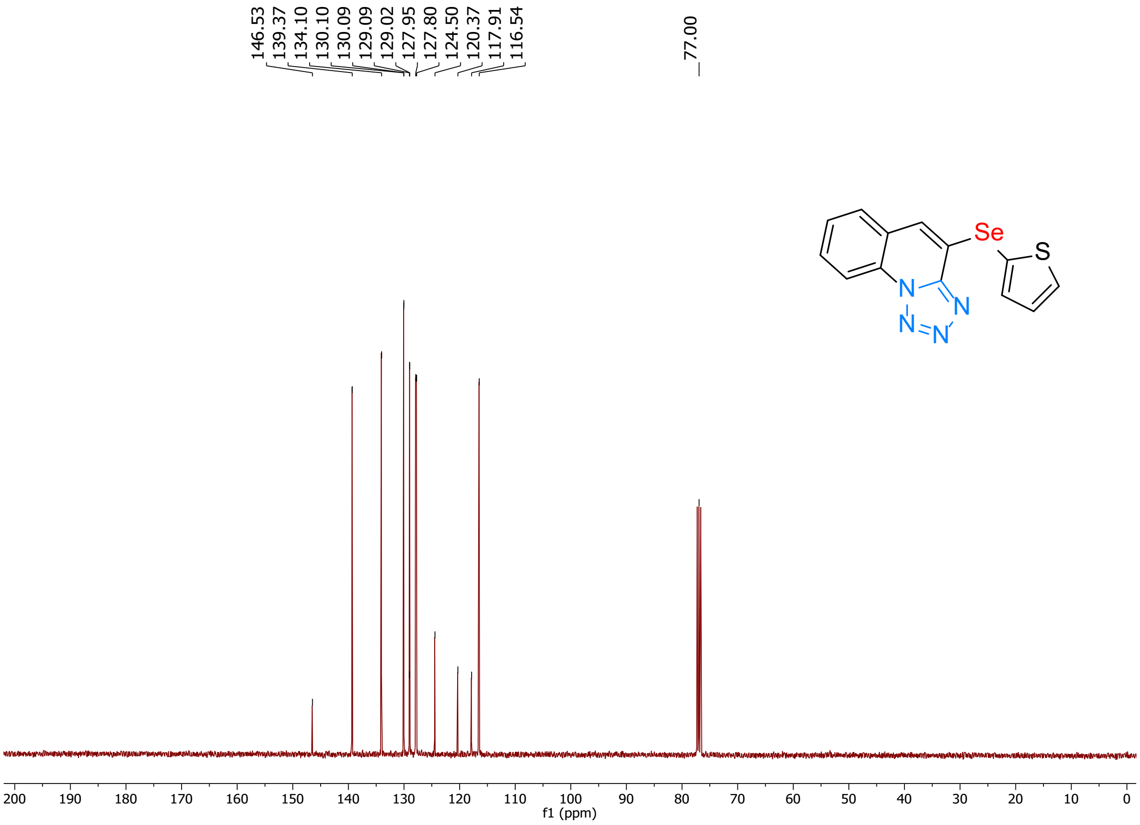

Figure S23. ^{13}C NMR (100 MHz) spectrum for compound **3h** in $CDCl_3$.

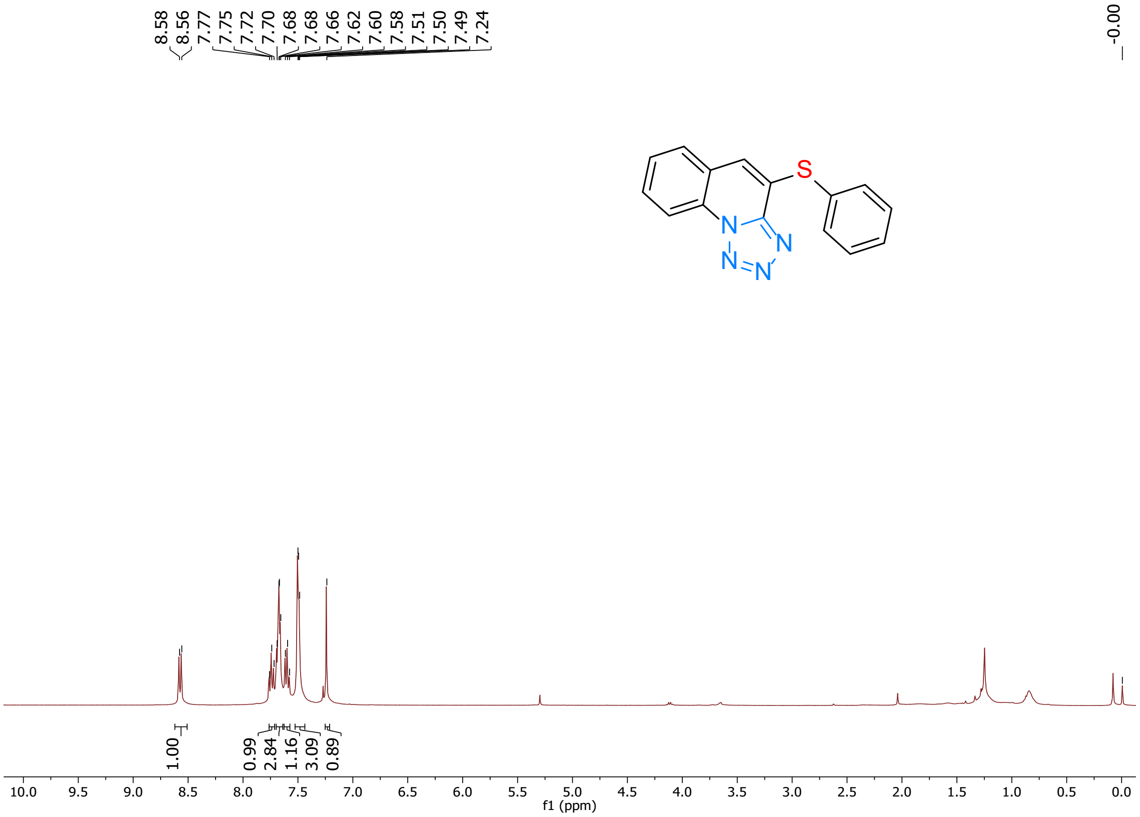

Figure S24. ^{1}H NMR (400 MHz) spectrum for compound **3i** in $CDCl_3$.

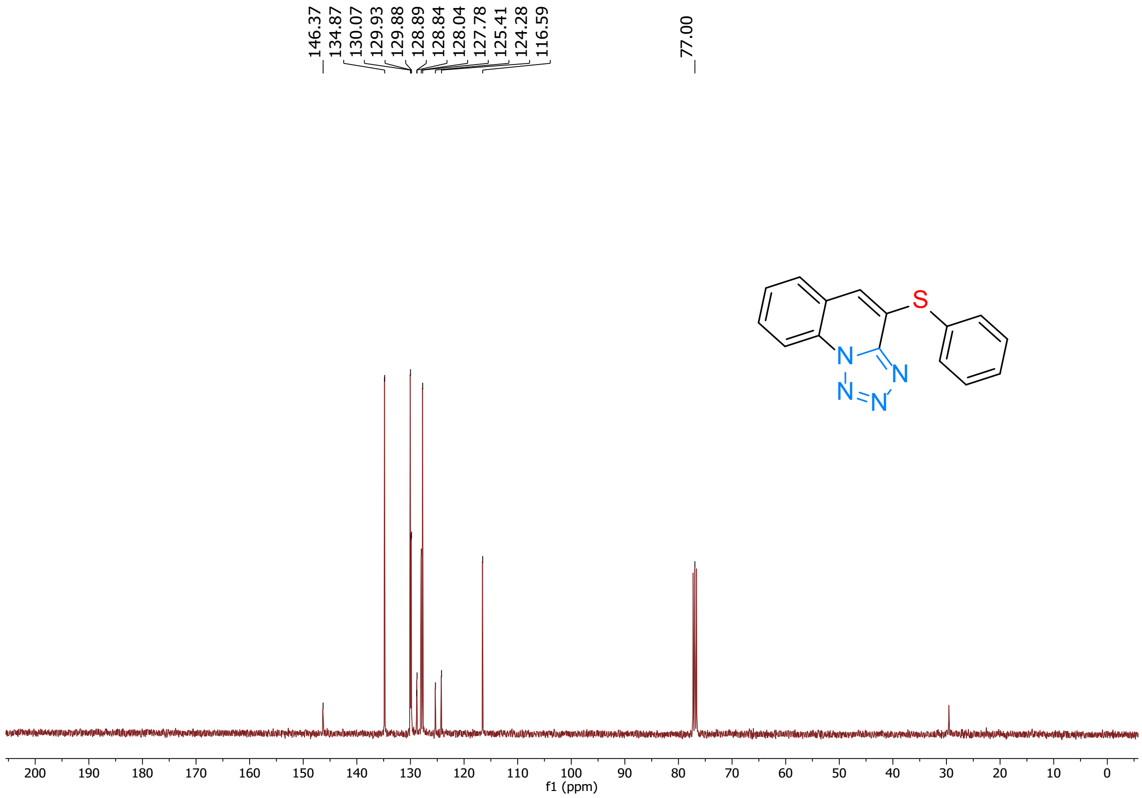

Figure S25. ^{13}C NMR (100 MHz) spectrum for compound **3i** in $CDCl_3$.

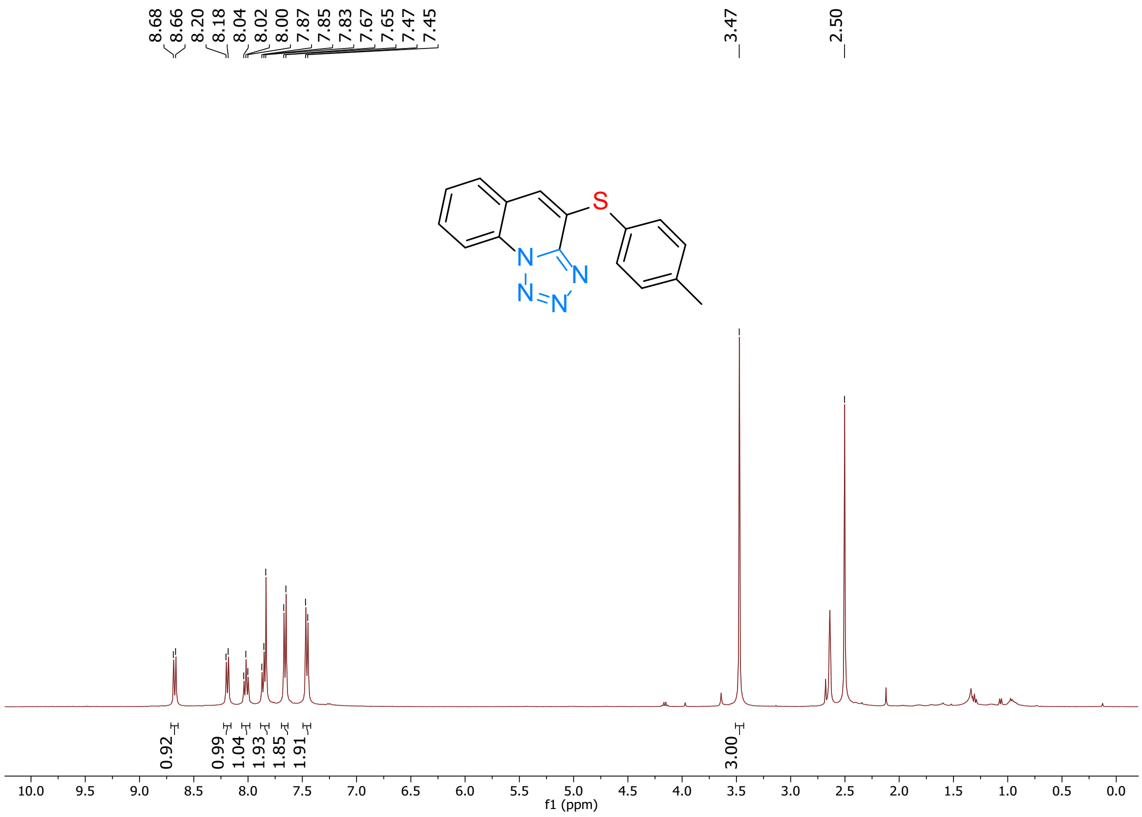

Figure S26. ^{1}H NMR (400 MHz) spectrum for compound **3j** in $DMSO_{d\text{-}6}$.

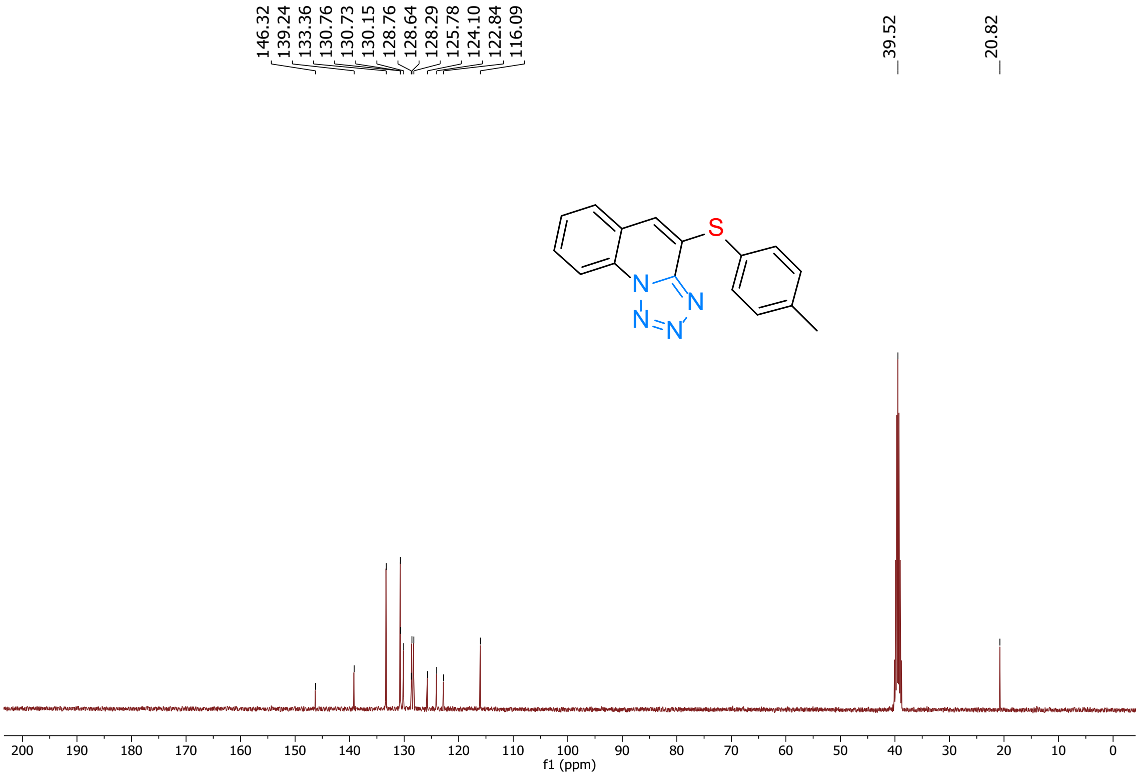

Figure S27. ^{13}C NMR (100 MHz) spectrum for compound **3j** in $DMSO_{d-6}$.

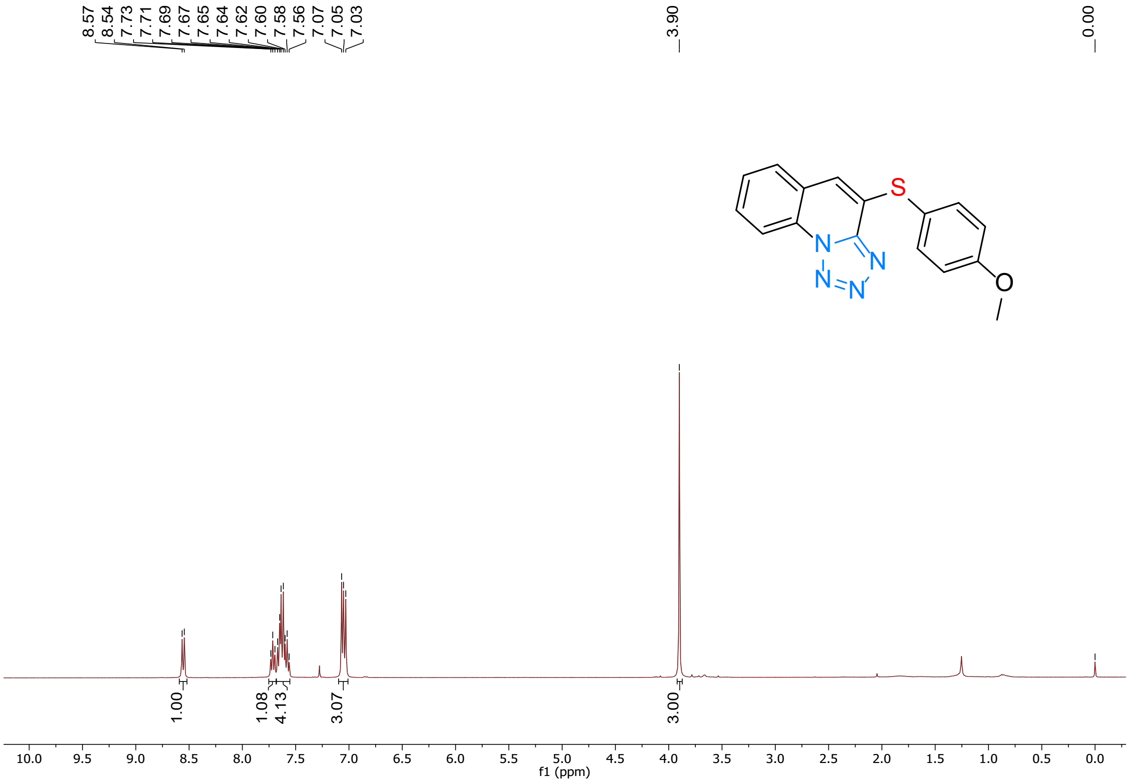

Figure S28. ^{1}H NMR (400 MHz) spectrum for compound **3k** in $CDCl_3$.

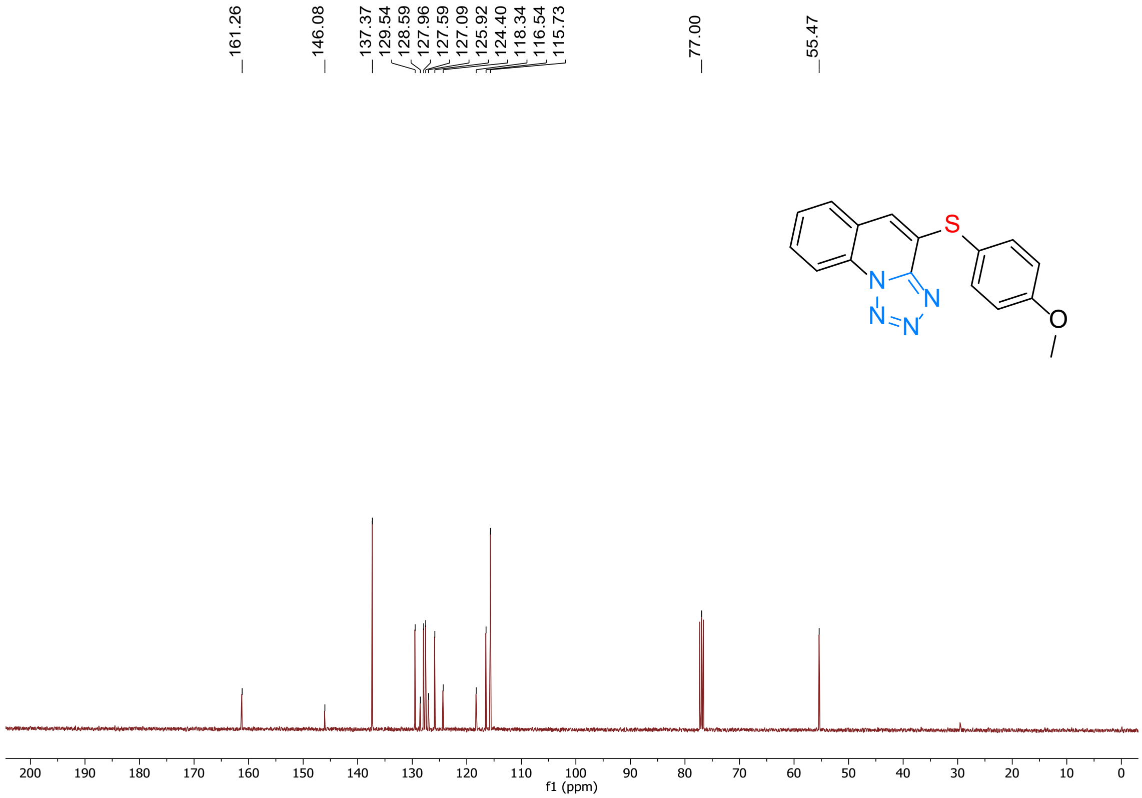

Figure S29. ^{13}C NMR (100 MHz) spectrum for compound **3k** in $CDCl_3$.

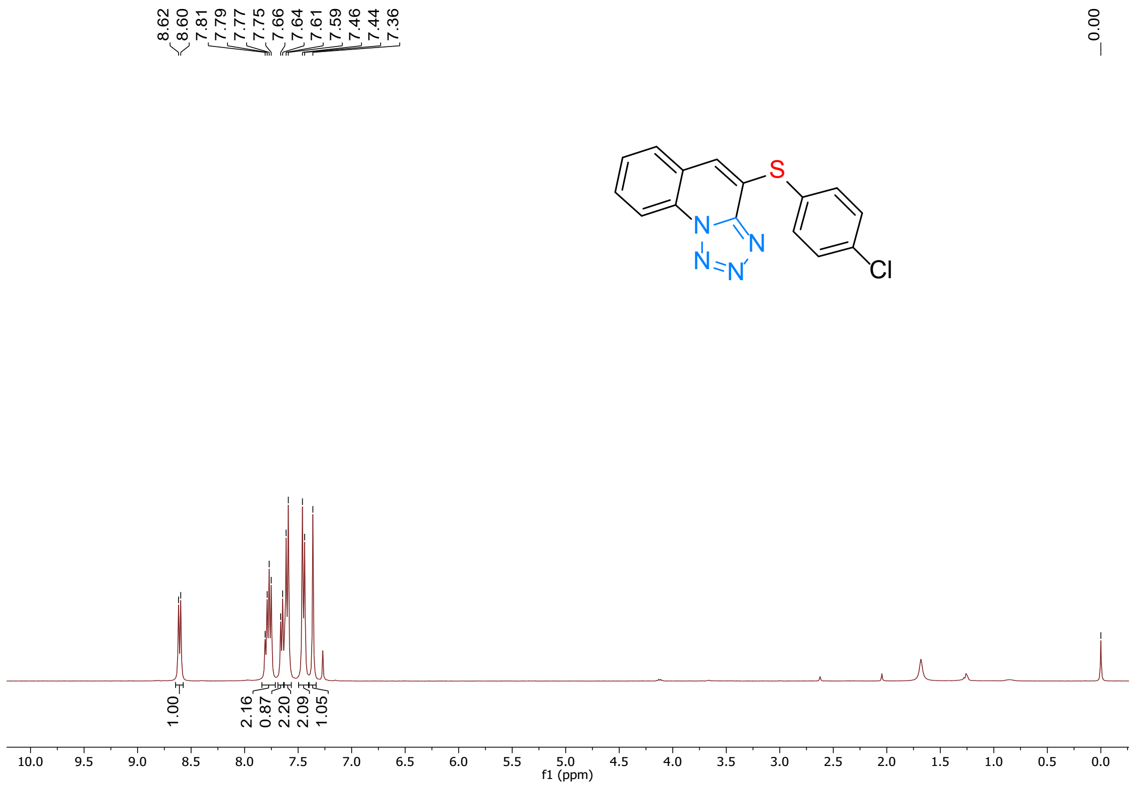

Figure S30. ^{1}H NMR (400 MHz) spectrum for compound **3l** in $CDCl_3$.

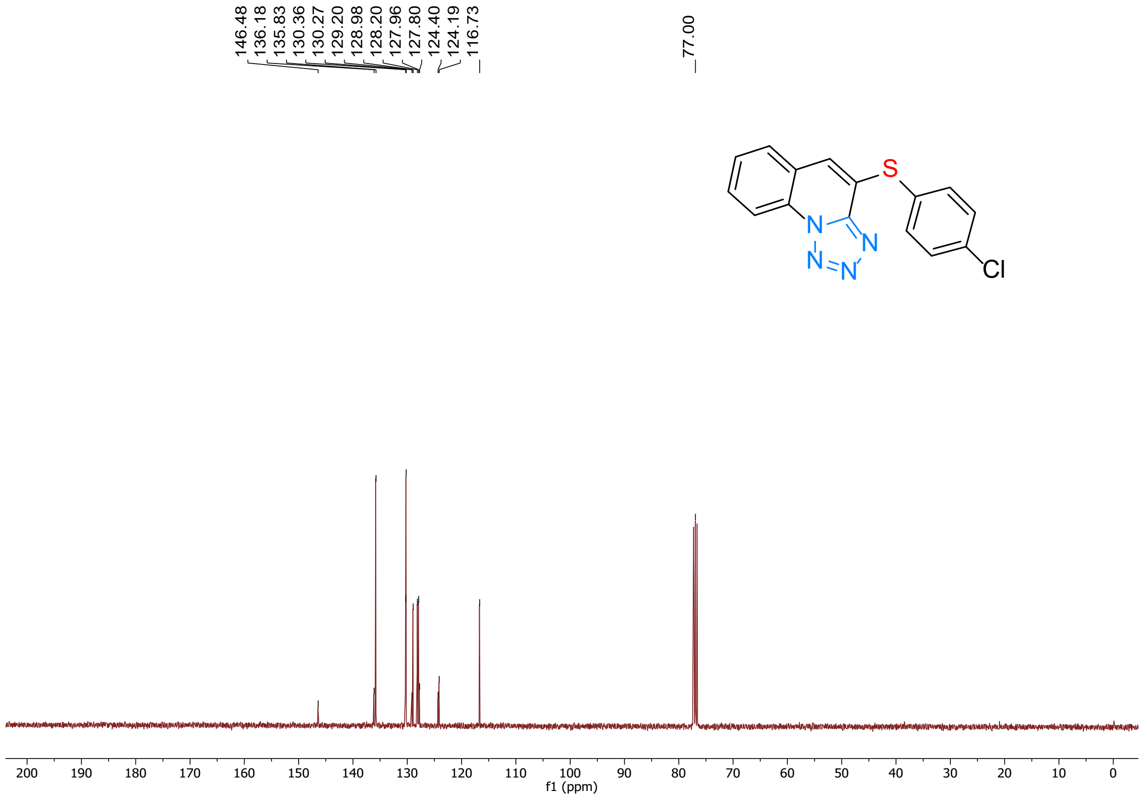

Figure S31. ^{13}C NMR (100 MHz) spectrum for compound **3l** in $CDCl_3$.

X-ray Crystallography:

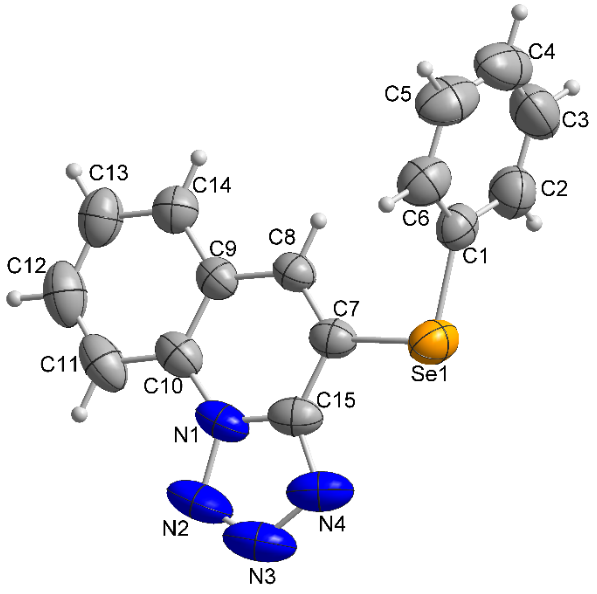

Figure S32. Thermal ellipsoid plot at the 50% probability level for the compound **3a**.

Table S1. Crystal data and structure refinement for compound **3a**.

Compound	**3a**
Formula	$C_{15}H_{10}N_4Se$
F.W. (g mol^{-1})	325.23
T (K)	296(2)
Crystal system	Monoclinic
Space group	$P2_1/c$
a (Å)	17.302(4)
b (Å)	5.1533(10)
c (Å)	14.968(3)
α (°)	90
β (°)	92.980(14)
γ (°)	90
V (Å^3)	1332.8(5)
Z	4
D_{calc} (g cm^{-3})	1.621
Abs. coef. (mm^{-1})	3.766
λ (Å)	1.54178
F (000)	648
Refl. collected	13605
Refl. unique *(R_{int})*	2634 (0.0285)
R_1 $[I > 2\sigma(I)]$	0.0323
wR_2 [I > 2σ(I)]	0.0789
R_1 (all data)[a]	0.0366
wR_2 (all data)[b]	0.0822
Goodness-of-fit on F^2	1.058
Largest diff. peak and hole (e.Å^{-3})	0.352 and -0.600

[a]$R_1 = \sum||F_o|-|F_c||/\sum|F_o|$; [b]$wR_2 = \{\sum w(F_o^2-F_c^2)^2/\sum w(F_o^2)^2\}^{1/2}$

Selected High Resolution Mass Spectrometry (HRMS)

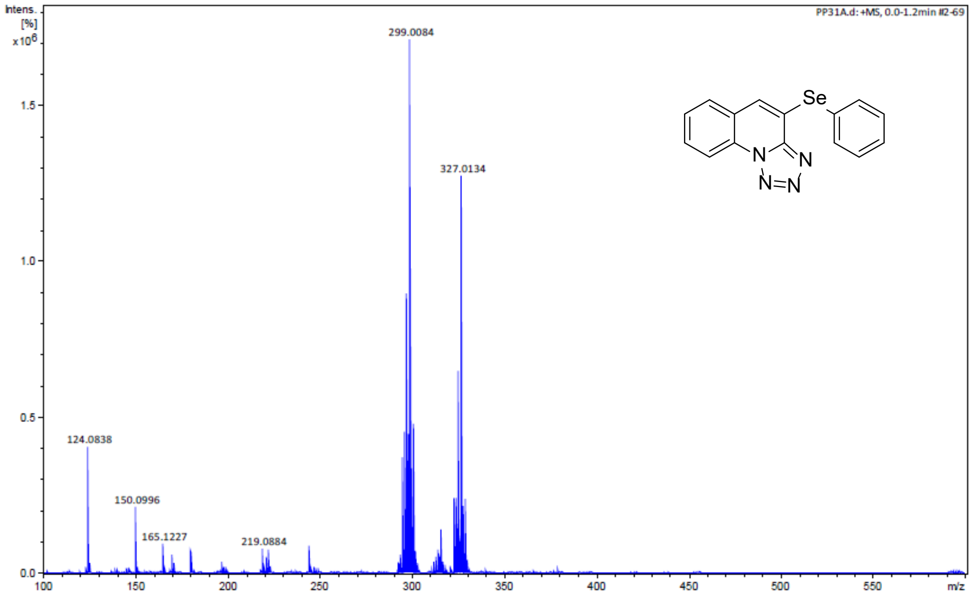

Figure S33. HRMS spectra of 4-(phenylselanyl)tetrazolo[1,5- *a*]quinoline.

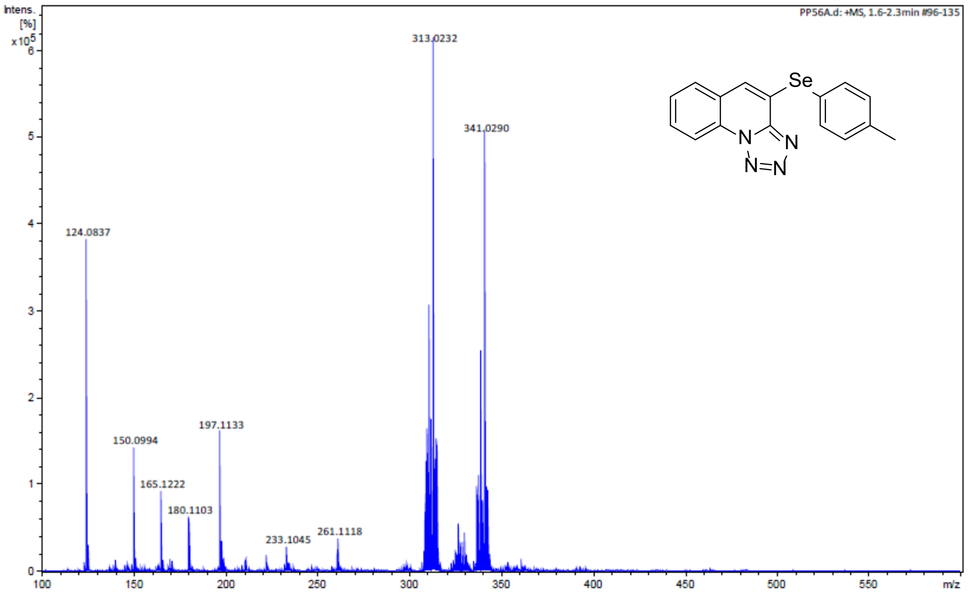

Figure S34. HRMS spectra of 4-(*p*-tolylselanyl)tetrazolo[1,5-*a*]quinoline.

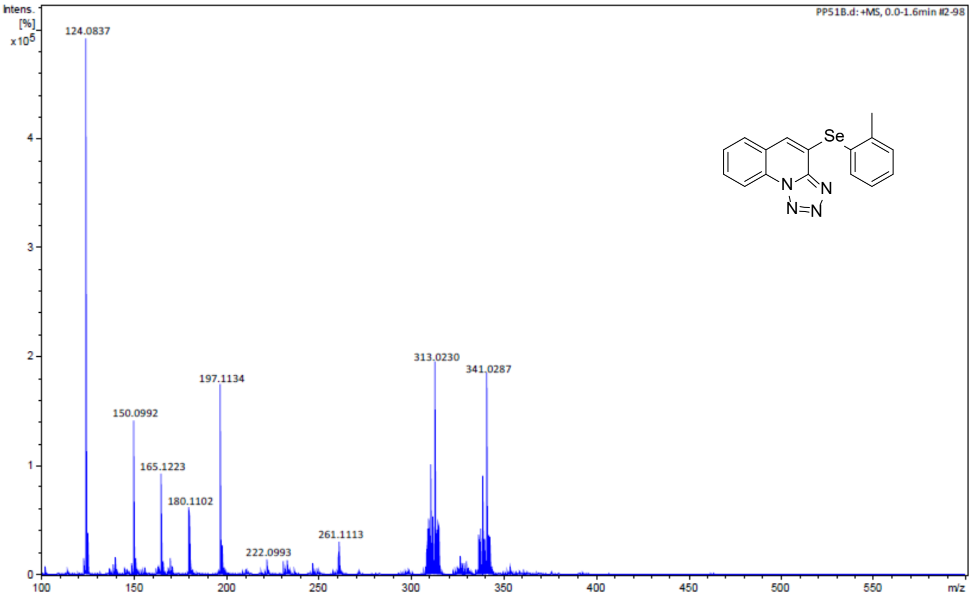

Figure S35. HRMS spectra of 4-(*o*-tolylselanyl)tetrazolo[1,5-*a*]quinoline.

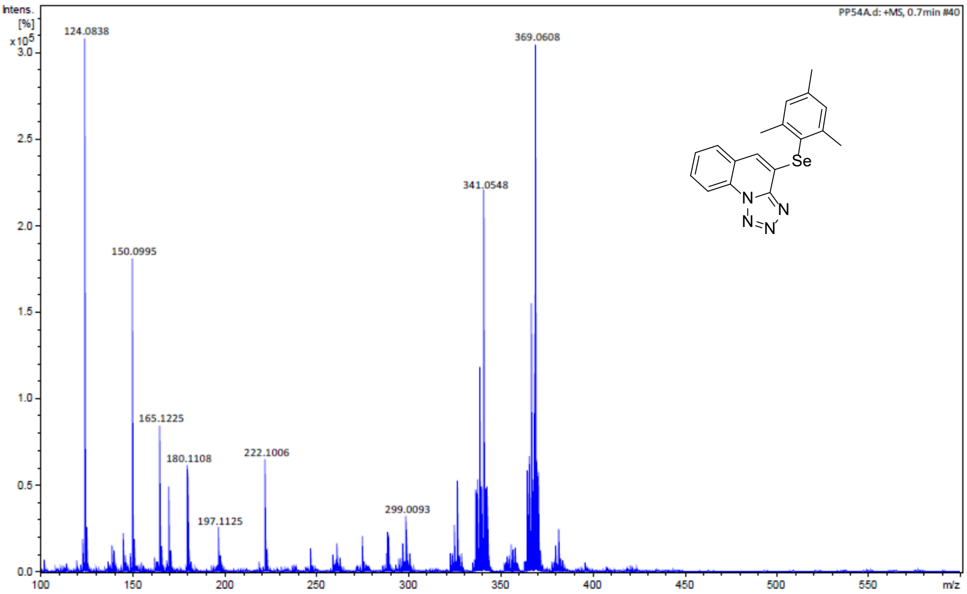

Figure S36. HRMS spectra of 4-(mesitylselanyl)tetrazolo[1,5-*a*]quinoline.

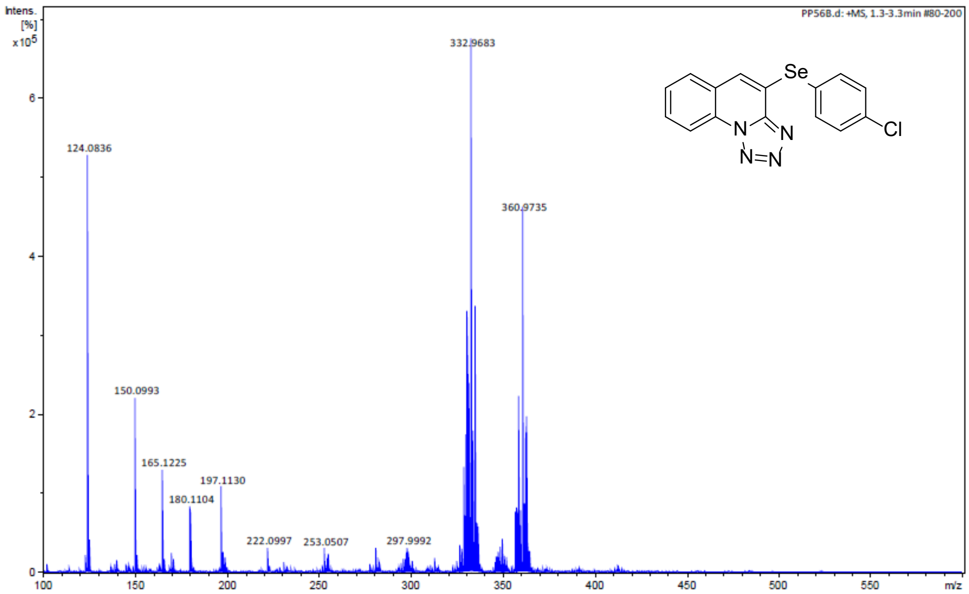

Figure S37. HRMS spectra of 4-((4-chlorophenyl)selanyl)tetrazolo[1,5-*a*]quinoline.

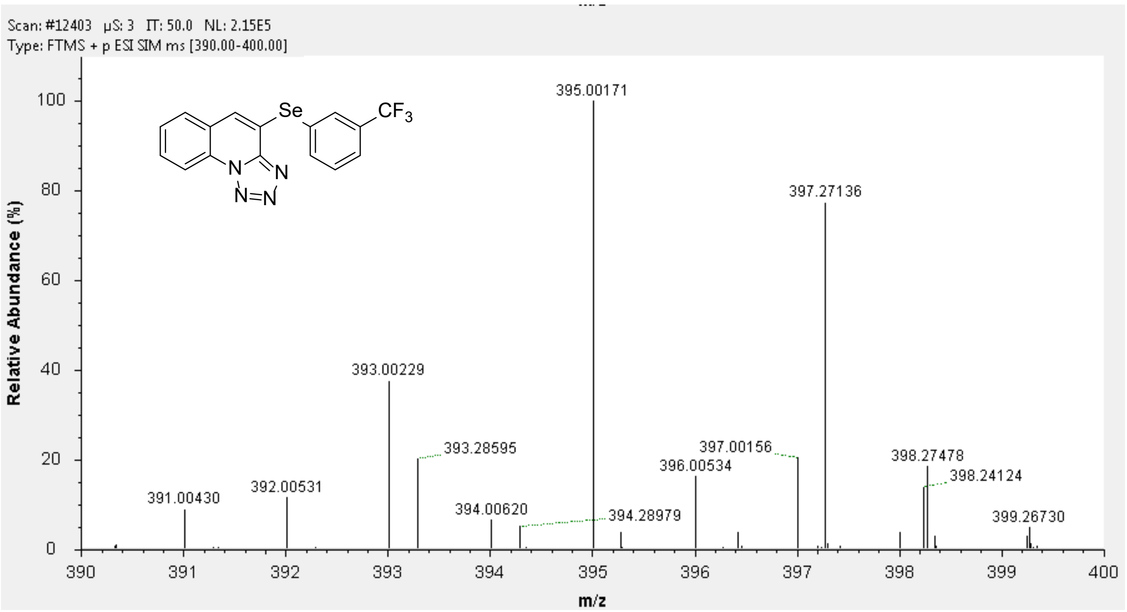

Figure S38. HRMS spectra of 4-((3-(trifluoromethyl)phenyl)selanyl)tetrazolo[1,5-*a*]quinoline.

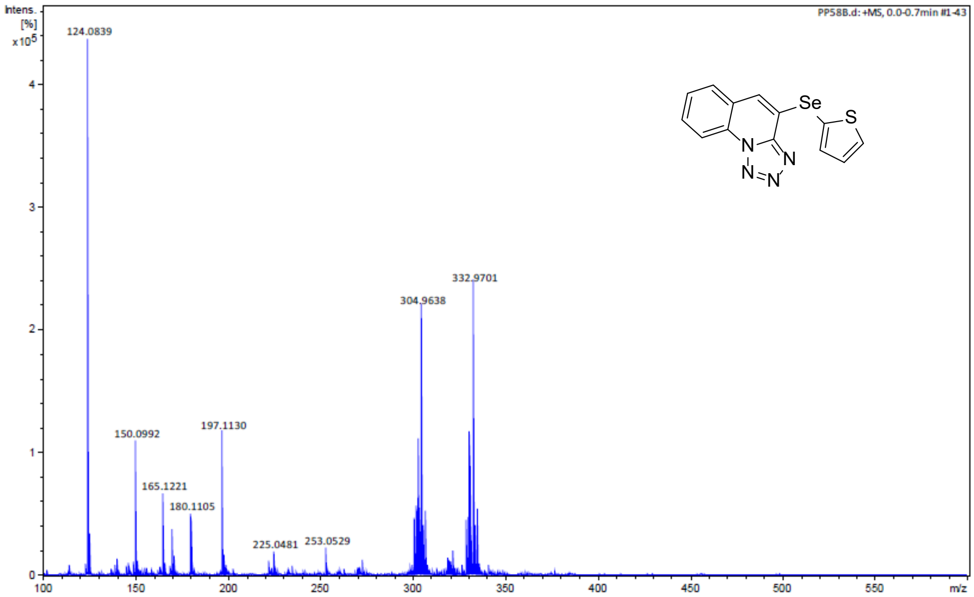

Figure S39. HRMS spectra of 4-(thiophen-2-ylselanyl)tetrazolo[1,5-*a*]quinoline.

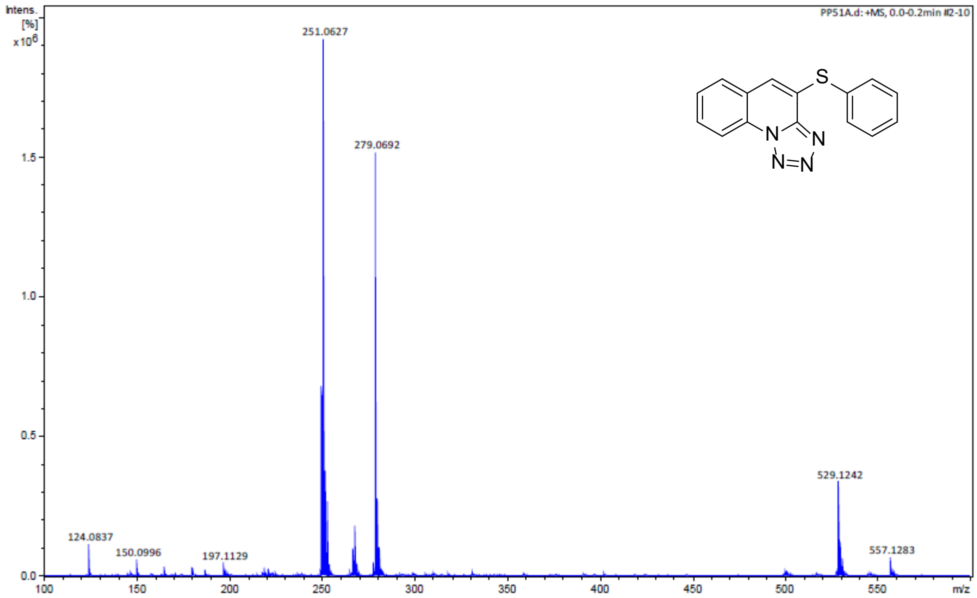

Figure S40. HRMS spectra of 4-(phenylthio)tetrazolo[1,5-*a*]quinoline.

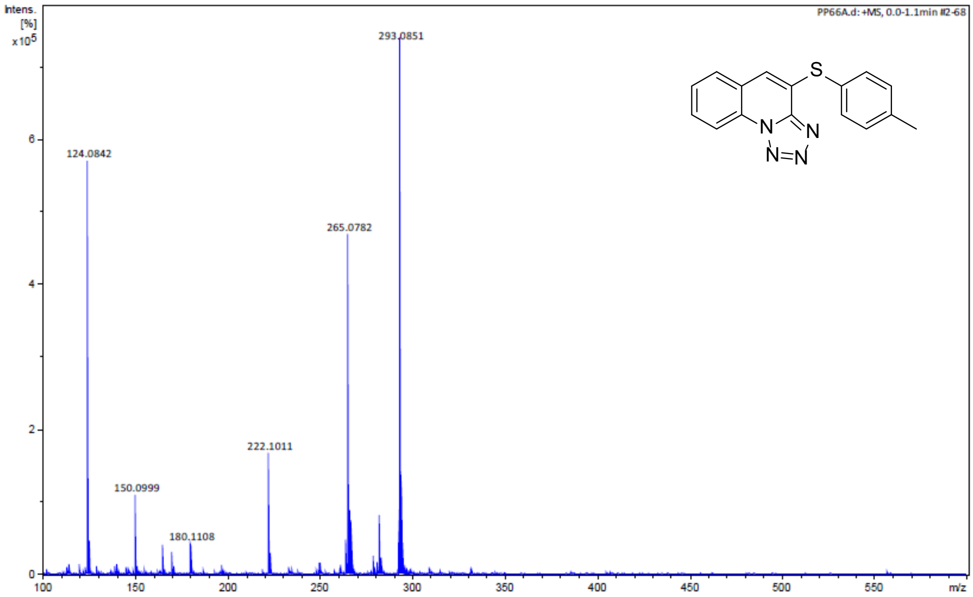

Figure S41. HRMS spectra of 4-(*p*-tolylthio)tetrazolo[1,5-*a*]quinoline.

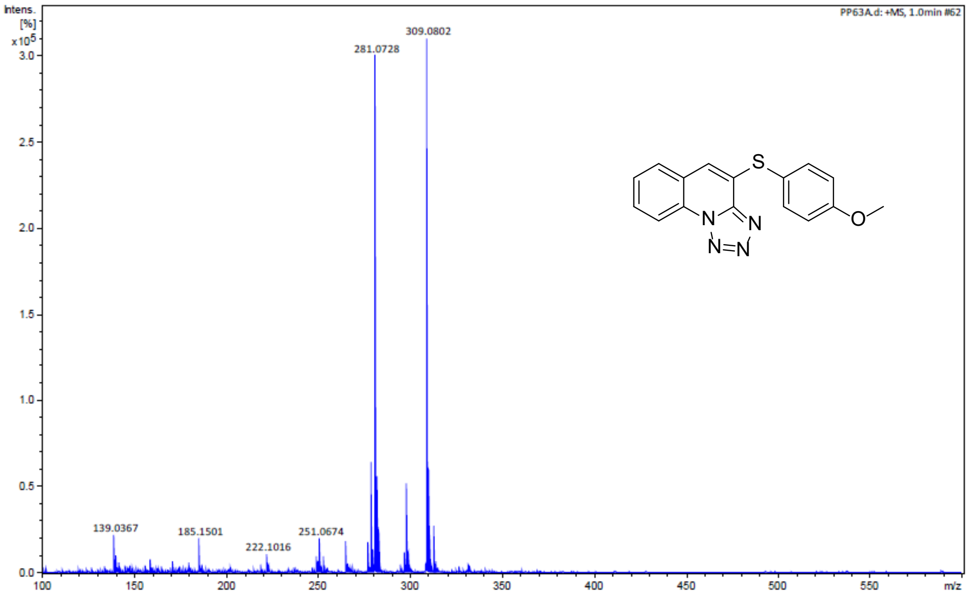

Figure S42. HRMS spectra of 4-((4-methoxyphenyl)thio)tetrazolo[1,5-*a*]quinoline.

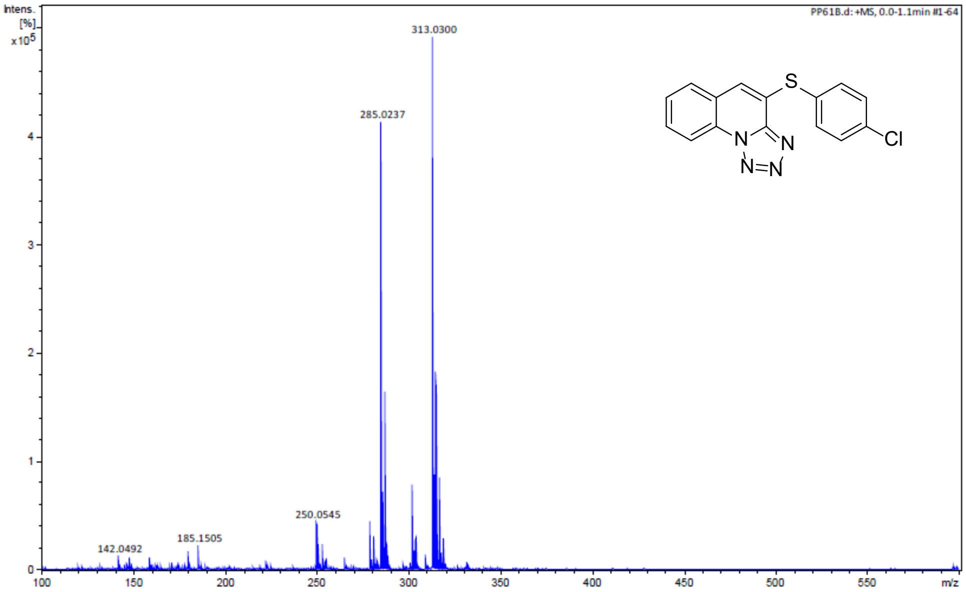

Figure S43. HRMS spectra of 4-((4-chlorophenyl)thio)tetrazolo[1,5-*a*]quinoline.
